# Incidence of concomitant illnesses in pregnancy in Indonesia: Estimates from 1990–2019, with projections to 2030

**DOI:** 10.1016/j.lanwpc.2021.100139

**Published:** 2021-04-13

**Authors:** Lareesa M Ryan, Mohammad A Mahmood, Caroline O Laurence

**Affiliations:** a School of Public Health, University of Adelaide, Adelaide, SA 5005, Australia; b Faculty of Medicine, Universitas Airlangga, Surabaya, Indonesia

## Abstract

**Background:**

‘Indirect’ causes of maternal death including concomitant illnesses such as infectious and non-communicable diseases (NCDs), accounted for 23% of maternal deaths in Indonesia in 2010. Reproductive-age women in Indonesia face a “double burden” of disease with increasing rates of NCDs and persisting rates of infectious disease. However, there is a lack of data on the burden of these diseases in pregnancy. The aim of this study was to estimate incidence of concomitant illnesses among pregnant women in Indonesia from 1990–2030.

**Methods:**

Publicly available data was accessed including incidence of concomitant illnesses in Indonesian reproductive-age women, population data and crude birth rate data from 1990–2019, and formed basis for projections to 2030. A dataset of estimates for all variables was generated for each year and sampled from a binomial distribution. Using these estimates, pregnancy estimates and incidence in pregnant women were calculated. A cubic splines model was fitted to generate estimates of incidence of concomitant illnesses in pregnancy.

**Findings:**

Past trends to 2019 show a decline in incident cases of infectious diseases except for HIV/AIDs, and an increase in most NCDs. In 2019, the most common disease was sexually transmitted infections. From 2020–2030, incidences of diabetes and lower respiratory infections are estimated to continue to increase.

**Interpretation:**

With an increasing incidence of NCDs and high-incidence of infectious diseases in pregnancy, Indonesian policymakers and stakeholders should consider what evidence-based strategies and interventions are best to reduce potential impacts of concomitant illnesses on pregnancy outcomes.

**Funding:**

Australian Government Research Training Program Scholarship.

**Research in context**

**Evidence before this study**

PubMed was searched for articles using the terms “incidence”, “pregnancy”, “Indonesia”, and by terms for concomitant illnesses singularly. This included no time restrictions and was not limited to English. This yielded no results for estimates at a national level. Grey literature was also searched using Google and Google Scholar, including United Nations (UN) agency health reports, the World Health Organization (WHO) Global Health Observatory database, and Indonesia Ministry of Health reports. Previously published estimates of concomitant illnesses in pregnancy in Indonesia were restricted to specific diseases in pregnancy, such as anaemia, HIV/AIDs, and malaria, and are limited to specific points in time. Estimates of tuberculosis were found during a previous literature search of the burden of indirect causes in low and middle income countries (LMIC) globally. In 2020, Global Burden of Disease (GBD) collaborators reported incidence estimates of concomitant illnesses in reproductive aged women (aged 15-49) in Indonesia across a broad range of infectious and non-infectious diseases from 1990 to 2019. However, estimations are not available among pregnant women. This determined the selection of using GBD data, and applying methods by Sugarman and colleagues to generate estimates in pregnancy. Searches were conducted from March 2019 to October 2020.

**Added value of this study**

To our knowledge, this study is one of the first to estimate incidence of concomitant illnesses in pregnancy in Indonesia over an extensive time period for a broad range of diseases. Where data are limited at a national level in Indonesia, these findings may help direct the focus of practice and policy in readiness of the health system to deal the with pre-existing issue of a high burden of infectious diseases in pregnancy, as well as the emerging problem of NCDs in pregnancy.

**Implications of all the available evidence**

Despite declines in maternal mortality in Indonesia, maternal care and the health system still face access and quality of care challenges, with some health facilities still lacking the capacity to deal with ‘direct’/obstetric causes of maternal mortality, such as preeclampsia, haemorrhage, and obstructed labour. This is a concern with rising rates of NCDs and pertaining high rates of infectious diseases as to what impact this may have on the health systems capacity to provide care for all complications and illnesses for pregnant women in Indonesia. Therefore, having an understanding of the burden of concomitant illnesses in pregnancy in Indonesia may help inform policy, strategies, and interventions to address the potential impact of concomitant illnesses on poor pregnancy outcomes and maternal mortality.

Alt-text: Unlabelled box

## 1 Introduction

In LMICs, including Indonesia, maternal mortality persists as a global health problem despite declines over the last two decades. In 2015, 303,000 maternal deaths occurred, with an estimated 99% occurring in LMICs [1]. In LMICs, it was estimated that the average maternal mortality rate (MMR) per 100,000 live births in 2015 was 239, significantly higher than compared to an average of 12 in high income countries (HICs) [2]. To address this issue the UN Sustainable Development Goals of 2030 included an aim to reduce the global MMR to less than 70 by 2030 [3]. Concomitant illnesses in pregnancy, also known as ‘indirect causes’ (non-obstetric complications), are one of the leading causes of poor pregnancy outcomes. Concomitant illnesses in pregnancy include infectious diseases such as tuberculosis, dengue fever, and HIV/AIDs, as well as NCDs such as heart disease and diabetes [4]. Some infectious diseases may increase in severity as the gestation progresses, or may lead to poor pregnancy outcomes [5]. For example, tuberculosis is linked to poor pregnancy outcomes such as premature birth, spontaneous abortion, intrauterine growth retardation and increased risk of perinatal death [6,7]. NCDs such as pre-existing diabetes are also associated with poor pregnancy outcomes, leading to an increased risk of complications such as pre-eclampsia, postpartum haemorrhage, and labour difficulties [8].

There is growing evidence of the contribution of concomitant illnesses to poor pregnancy outcomes. From 2003 to 2009, Say et al. reported that concomitant illnesses (including pre-existing medical conditions and HIV-related deaths) accounted for 20.3% (491 000) maternal deaths in LMICs [9]. From 2007 to 2017, the GBD Cause of Death Collaborators also observed that most direct causes of maternal death have declined steadily (haemorrhage by 52.1%, sepsis and other infections by 27.1% and obstructed labour by 17.7%) but indirect causes of maternal death (excluding HIV/AIDS) have declined only marginally by 4.1%. This ranked indirect causes as the second leading cause of maternal death in 2017, with 34,100 deaths [10]. In addition, a secondary analysis of the WHO Multi-Country Survey on Maternal and Newborn Health analysed severe maternal health outcomes (near miss of maternal death and maternal mortality) of 314,623 pregnancies across 28 LMICs in Africa, Asia, Latin America, and the Middle East, plus Japan (HIC) [11]. The study noted that a total of 2822 women had severe maternal health outcomes. Of these women, indirect causes occurred in 20.9% of these cases [11].

In Indonesia, the MMR remains high at 177 deaths per 100,000 livebirths in 2017 [12]. In the same year, Indonesia recorded 5.3 million pregnancies, and 4.84 million livebirths [13,14]. In 2017, Indonesia had an estimated population of 264 million, the fourth largest populated country in the world with an estimated number of 69.6 million women of reproductive age (15–49 years old) [13], [14], [15]. At a national level, Mboi et al [16]. noted that Indonesians, including reproductive aged women, face a “double-burden” of disease, with epidemiological trends from 1990 to 2016 highlighting the persisting burden of infectious diseases such as tuberculosis, HIV/AIDs, and diarrhoeal diseases, as well as an increasing burden of NCDs, including heart disease, and diabetes. A follow-up study of the 2010 Indonesian Population Census that analysed pregnancy-related deaths also reported that 22.8% of maternal deaths were due to indirect causes with the leading causes being circulatory diseases, tuberculosis, respiratory diseases, and other specified infectious and parasitic diseases [17].

Whilst some data are available regarding estimates of concomitant illnesses among Indonesian pregnant women at points in time, such as for tuberculosis [6], HIV [18], and anaemia [18], as well as among reproductive aged Indonesian women [19], limited data are available on past, current, and future incidence of a broader range of concomitant illnesses among pregnant women. The aim of this study was to estimate the past and future burden of concomitant illnesses in pregnancy for Indonesian women from 1990 to 2030 using publicly accessible data.

## 2 Methods

### 2.1 Study population

The study population included pregnant women aged 15–49 years old in Indonesia who experienced a concomitant illness. The inclusion and exclusion criteria for selection of concomitant illnesses in pregnancy are outlined below under the heading data analysis.

### 2.2 Data sources

To estimate the incidence of concomitant illnesses among pregnant women in Indonesia, publicly available data were utilised. This included total population and population of reproductive aged women (15–49 years old) data accessed from GBD Collaborative Network [15], crude birth rate data accessed from The World Bank [20], and the estimated incidence of concomitant illnesses among reproductive aged women (15–49 years) from the GBD Collaborative Network [19]. Incidence data on concomitant illnesses was extracted using the GBD Results tool, corresponding to the Level 3 hierarchy of disease and injury classification. Data utilised from the GBD Collaborative Network was available for all years from 1990 to 2019. Data from The World Bank was available from 1990 to 2018, therefore crude birth rate data in 2019 was based on 2018 data. Information on the data input sources for the GBD Collaborative Network estimates are available from the GBD Result tool on the Global Health Data Exchange website [19]. This observed data from 1990 to 2019 formed the basis for projections from 2020 to 2030.

### 2.3 Data analysis

Concomitant illnesses included for analysis were selected on the basis of their potential impact on poor maternal health outcomes. Diseases were further excluded on the basis of low number of incident cases (of <1000 cases) among Indonesian reproductive aged women (15–49 years old).

A simulation used the values for population of women aged between 15 and 49 years, birth rate, and incidence of disease for each year to generate a dataset of 100 estimates for each year. For each of these three variables the value for each year was converted to a proportion and then 1000 samples were taken from a binomial distribution based on this proportion. The average across the 1000 samples was used as the proportion for that estimate. This process was repeated 100 times. Using these 100 estimates per year, pregnancy estimates and incidence of disease in pregnant women were calculated using the formulae described by Sugarman and colleagues [6]. A cubic splines model [21] was then fitted to this data to generate estimates of incidence of the disease in pregnant women aged 15–49 years of age in Indonesia at a country level for each year. This model assumes that population growth, pregnancy rates, and incidence of concomitant illnesses in reproductive aged women in Indonesia will follow similar trends based on observed data values. All analysis was completed using STATA/IC 15•1 and Microsoft Excel 2013. A more detailed description of the statistical analysis, model, its assumptions and formulas is provided in the supplementary file. Results reported in the main text includes the 22 most prevalent concomitant illnesses. Annual estimates from 1990 to 2030 for all diseases are accessible in the appendix. This study has been reported in compliance with the Guidelines for Accurate and Transparent Health Estimates Reporting (GATHER)[22], as described in the supplementary material file.

### 2.4 Role of the funding source

This study was supported by an Australian Government Research Training Program (RTP) Scholarship. The funder of the study had no role or involvement in this study. The corresponding author had full access to all of the data and the final responsibility to submit for publication.

## 3 Results

### 3.1 Past estimates: 1990–2019

The incident estimates for concomitant illnesses in pregnancy in Indonesia were grouped into infectious diseases and NCDs. The total cases (95% CI) in pregnant women from 1990 to 2019 and mean annual cases (95% CI) from 1990 to 2019 are presented in Table 1. In addition, Table 1 presents the mean percentage change over time from 1990 to 2019, and the mean annual percentage change (95% CI) across ten-year intervals from 1990 to 2019.

**Table 1:** Total incident cases, mean annual cases, mean annual percentage change (by time periods), and percentage over time of concomitant illnesses in pregnancy in Indonesia, 1990–2019.

Concomitant illnesses with GBD codes	Total incident cases in pregnant women	Mean annual cases (95% CI)	Mean annual percentage (%) change (95%CI)*			Percentage (%) change over time 1990-2019 (95% CI)#
**Time period**	1990–2019	1990–2019	1990–1999	2000–2009	2010–2019	1990–2019
**Infectious diseases**						
Acute hepatitis A•5•8	3068809 (3000134 to 3137484)	102294 (100004 to 104583)	-0.5 (-0.4 to 0.7)	0.3 (0.3 to 0.3)	-2.7 (-3.1 to -2.4)	-28.9(0.9 to -30.2)
Dengue A•4•11	1198701 (1163081 to 1234320)	39957 (38769 to 41144)	-2.4 (-2.3 to -2.5)	21.8 (1.9 to 1.8)	-1.8 (-2.2 to -1.4)	-24.2 (0.9 to -25.5)
HIV/AIDs A•1•1	4115 (2403 to 5828)	137 (80 to 194)	20.7 (-21.0- to 2.6)	11.5 (22.4 to 8.1)	-2.7 (-9.9 to 0.3)	92.8 (0.9 to 190.9)
Lower respiratory infections A•2•2	3612672 (3533299 to 3692045)	120422 (117777 to 123068)	-0.9 (-0.9 to -1.0)	-2.4 (-2.4 to -1.0)	1.2 (0.9 to 1.4)	-23.3 (-0.9 to -24.2)
Malaria A•4•1	824950 (793504 to 856396)	27498 (26450 to 28547)	-6.2 (-6.2 to -6.1)	-4.9 (5.0 to 4.7)	-57.8 (1872.1 to -35.5)	-2573.3 (-0.9 to 123374.2)
Measles A•5•6	64917 (58081 to 71753)	2164 (1936 to 2392)	-2.0 (-1.7 to -2.3)	-7.8 (-8.7 to -7.0)	-3.0 (-6.8 to -0.7)	-247.7 (0.9 to -385.2)
Sexually transmitted infection excluding HIV/AIDs A•1•2	16400217 (16104353 to 16696080)	546674 (536812 to 556536)	-0.5 (-0.4 to -0.6)	1.5 (1.5 to 1.4)	-1.2 (-1.5 to 1.0)	-1.6 (0.9 to -1.7)
Tuberculosis A•2•1	241818 (228078 to 255557)	8061 (7603 to 8519)	-6.3 (-6.4 to -6.3)	-5.6 (-5.9 to -5.3)	-7.6 (-10.8 to -5.4)	-544.5 (0.9 to -742.7)
**Non communicable diseases**						
**Cardiovascular diseases**						
Cardiomyopathy and myocarditis B•2•6	10117 (7420 to 2814)	337 (247 to 427)	-0.4 (2.1- to -1.5)	0.6 (0.8 to 0.5)	-2.6 (-9.3 to 0.3)	-21.8 (0.9 to -45.3)
Ischemic heart disease B•2•2	14019 (10848 to 17190)	467 (362 to 573)	0.1 (2.3 to -1.0)	0.5 (0.6 to 0.4)	-3.4 (-9.2 to -0.5)	-25.4 (0.9 to -47.7)
Non-rheumatic valvular heart disease B•2•5	1725 (643 to 2807)	58 (21 to 94)	12.2 (165.0 to 2.7)	-3.0 (-6.2 to -1.9)	3.9 (-7.4 to 5.9)	68.2 (0.9 to 324.8)
Stroke B•2•3	100911 (92280 to 109543)	3364 (3076 to 3651)	0.1 (0.8 to -0.5)	2.4 (2.6 to 2.3)	-1.6 (-2.8 to 0.6)	10.6 (0.9 to 12.1)
**Endocrine disorders**						
Chronic kidney disease B•8•2	61335 (5459 to 68071)	2044 (1820 to 2269)	1.7 (3.2 to 0.7)	3.0 (3.4 to 2.7)	-0.1 (-1.3 to 0.9)	35.9 (0.9 to 42•2)
Diabetes mellitus B•8•2	159874 (148882 to 170866)	5329 (4963 to 5696)	4.1 (5.3 to 3.2)	-1.0 (-1.1 to -1.0)	6.9 (6.8 to 7.0)	57.9 (0.9 to 62.1)
**Neoplasms**						
Breast cancer B•1•14	32903 (27997 to 37809)	1097 (933 to 1260)	1.5 (3.6 to 0.2)	4.1 (4.7 to 3.6)	-3.0 (-5.6 to -1.3)	23.8 (0.9 to 31.4)
Cervical cancer B•1•15	13344 (10257 to 6430)	445 (342 to 548)	0.6 (3.1 to -0.7)	0.3 (0.4 to 0.1)	-4.7 (-12.7 to -1.5)	-38.8 (0.9 to -83.3)
Ovarian cancer B•1•17	4578 (2802 to 6354)	153 (93 to 212)	4.1 (-38.0 to 0.4)	2.9 (4.7 to 2.1)	1.4 (-3.3 to 3.5)	54.7 (0.9 to 104.2)
**Mental health disorders**						
Anxiety B•6•4	953444 (922549 to 984339)	31781 (30752 to 32811)	-0.6 (-0.4 to -0.8)	1.2 (1.3 to 1.2)	-1.0 (-1.3 to -0.7)	-4.1 (0.9 to -4.3)
Bipolar B•6•3	45453 (39704 to 51202)	1515 (1323 to 1707)	0.7 (1.8 to -0.1)	0.1 (0.2 to 0.1)	-3.6 (-6.3 to -1.7)	-25.1 (0.9 to -34.3)
Depressive disorders B•6•2	2876017 (2810278 to 2941756)	95867 (93676 to 98059)	-1.0 (-0.9 to -1.1)	0.9 (0.9 to 0.9)	-0.6 (-0.9 to -0.4)	-6.3 (0.9 to -6.5)
**Malnutrition**						
Vitamin A deficiency A•7•3	11853474 (11626524 to 12080425)	395116 (387551 to 402681)	-5.2 (-5.2 to -5.2)	-3.7 (-3.8 to -3.6)	-6.4 (-7.1 to -5.8)	-323.5 (0.9 to -347.4)
**Respiratory disorders**						
Chronic obstructive pulmonary disease B•3•1	68634 (61550 to 5719)	2288 (2052 to 2524)	-0.7 (1.6 to 0.0)	1.2 (1.3 to 1.1)	-2.1 (-3.8 to -0.9)	-0.4 (0.9 to -0.7)

⁎ Mean APC was calculated on the percentage change for each year, then averaged across the 10 year time period.

# Calculated based on percentage change from 1990-2019

Over the period 1990 to 2019, Sexually Transmitted Infection's (STIs) were the most common infectious disease with an estimated 16,400,217 total incident cases, whilst HIV/AIDs were the least common infectious disease with an estimated 4115 total incident cases (Table 1). Of NCDs in pregnancy, Vitamin A deficiency was the most common with an estimated 11,853,474 incident cases, whilst non-rheumatic valvular heart disease was the least common with an estimated 1725 incident cases.

From 1990 to 2019, the mean percentage (%) change over time shows an increase in the incidence of most NCDs, including non-rhematic valvular disease (68.2%), and diabetes mellitus (57.9%). Comparatively, there was a decline of most infectious disease among Indonesian pregnant women, such as malaria (-2573.3%) and tuberculosis (-544.5%), with only the incidence of HIV/AIDs (92.8%) increasing during this period.

The mean annual percentage changes (APC) notably show an increase of incident cases of dengue (21.8%) during the period 2000 to 2009, and non-rheumatic heart valvular disease (12.2%) during the period 1990 to 1999. In contrast, there was a large decline in malaria (-57.8%) during the period 2010 to 2019 and Vitamin A deficiency (-6.4%) declined during the period 2010 to 2019.

### 3.2 Current estimates: 2019

In Table 2, the estimated incident cases from 2019 were ranked to identify the twelve most common concomitant illnesses across infectious and NCDs among pregnant women in Indonesia in 2019. In 2019, approximately 1 in 10 pregnant women were estimated to have an STI (excluding HIV/AIDs), 1 in 20 pregnant women had Vitamin A deficiency, 1 in 35 pregnant women had a lower respiratory infection and 1 in 50 pregnant women had a depressive disorder.

**Table 2:** Leading 12 concomitant illnesses in pregnancy in Indonesia by incident cases per 100 000 pregnant women in 2019.

Rank	Concomitant illnesses with GBD reference code	Incident cases per 100 000 pregnant women in 2019 (95%CI)
1	Sexually transmitted infection excluding HIV/AIDs A•1.2	13601 (13194–14008))
2	Vitamin A deficiency A•7.3	4533 (4221–4845)
3	Lower respiratory infections A•2.2	2958 (2849–3067)
4	Depressive disorders B•6.2	2422 (2331–2512)
5	Acute hepatitis A•5.8	2159 (2065–2254)
6	Dengue A•4.11	947 (898–996)
7	Anxiety B•6.4	787 (744–829)
8	Diabetes mellitus B•8.2	220 (205–235)
9	Stroke B•2.3	86 (74–98)
10	Tuberculosis A•2.1	71 (52–90)
11	Chronic Kidney Disease B•8.2	60 (51-70)
12	Chronic Obstructive Pulmonary Disease B•3.1	53 (44–63)

Infectious diseases  Non-communicable diseases

### 3.3 Projected estimates: 2020–2030

**Fig. 1:**
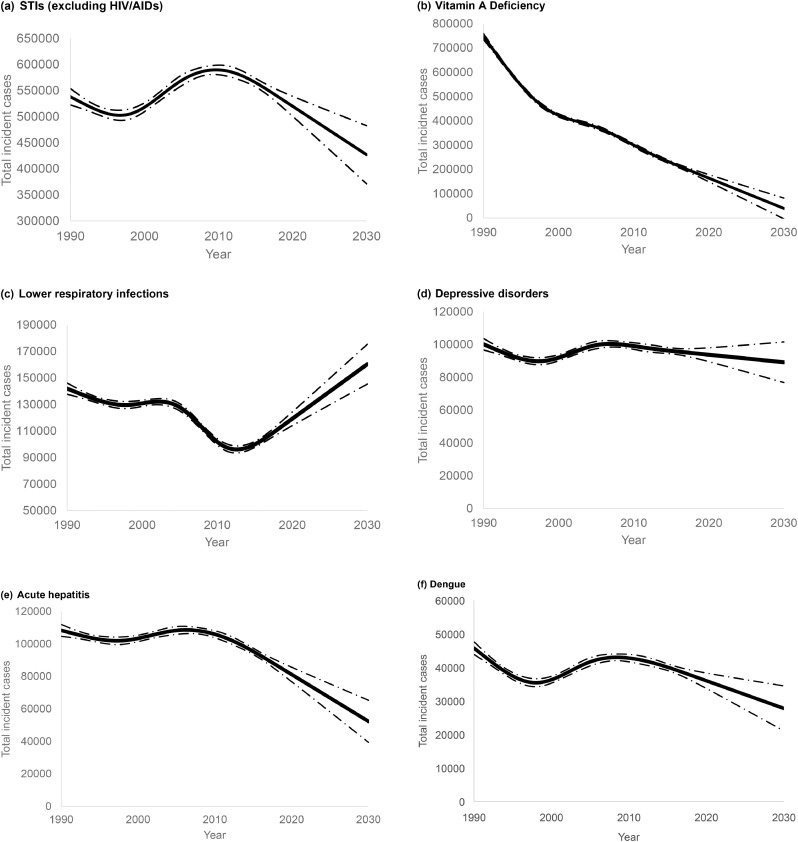
Projected trends of total incident cases (95%CI) in pregnancy in Indonesia to 2030, based on observed data 1990-2019, for leading 6 concomitant illnesses in pregnancy in 2019.

The projected estimates (2020 to 2030) for the six most common concomitant illnesses in 2019 are shown in Fig. 1. Projected estimates for all other concomitant illnesses are available in the appendix. It is estimated that over the projection period there will be an increase in incidence of diabetes mellitus, measles, non-rheumatic heart disease, lower respiratory infections, and some cancers in pregnancy, and a decrease in incidence of tuberculosis and malaria in pregnancy in Indonesia. Also, for other concomitant illnesses in pregnancy, such as depressive disorders, anxiety, Vitamin A deficiency, STIs, acute hepatitis, and dengue it is expected there will be a decrease in their incidence, although the number of incident cases among Indonesian pregnant women will still remain high.

## 4 Discussion

Overall, estimates observed and projected for the incidence of concomitant illnesses among Indonesian pregnant women present an increase in some NCDs such as diabetes mellitus, some cardiovascular diseases, and some cancers. For infectious diseases during pregnancy, incidence estimates mostly show a decline, with the exception of measles and lower respiratory infections.

Our analysis found some notable fluctuations of mean annual percentage changes within time periods for some diseases, such as HIV/AIDs and diabetes mellitus, which may be a reflection of particular events, such as epidemics among particular population groups [23],and changes to national survey monitoring and reporting measures respectively [24]. Nationally, Indonesia has also introduced routine screening of STIs in antenatal care [25], as well as of malaria in endemic areas [26], which may be an underlying factor for incident rate fluctuations.

This study has also shown the effect on incidence rates of programs targeting concomitant illnesses. The estimated incidence rates for tuberculosis, malaria, STIs (except HIV/AIDs), Vitamin A deficiency, and acute hepatitis are estimated to fall significantly by 2030. A contributory factor for this trend is likely to be the significant funding and health programs invested in the global health priorities of malaria and tuberculosis during pregnancy in LMICs that has occurred over the last two decades [27], [28], [29]. Introduction of successful treatments is another contributory factor, for example, Vitamin A supplementation has been shown to improve serum retinol levels of Vitamin A in pregnant women [30]. In addition, research has shown that Vitamin A supplementation has also contributed to the decline of maternal deaths in LMICs with high MMR (>500) and high prevalence of gestational night time blindness [31]. In contrast, the estimated rise in other diseases such as diabetes, may reflect to some extent the limited funding and programs to address these conditions both in the general population but also in pregnant women in LMICs.

The pattern of incidence rates for concomitant illnesses in pregnant women found in Indonesia reflects a country that is moving from a predominantly LMIC where rates of infectious diseases are high, to increasing rates of NCDs such as diabetes, a situation most commonly found in HICs, known as an epidemiological transition [9,32]. The past, current, and future estimates of the burden of concomitant illnesses in pregnancy in Indonesia supports the hypothesis of the “double burden” of disease in the general population of Indonesia that sees the persisting problem of infectious diseases in conjunction with an increase of NCDs [16,33].

In this time of epidemiological transition, the current national and international funding, strategies, and programs that focus on direct and some indirect causes of maternal deaths needs to be expanded to encompass NCDs in pregnancy in order make further reductions in MMR in Indonesia, like in many other LMICs [27], [28], [29]. This research suggests that this focus should now be extended to other concomitant illnesses that are estimated to increase such as, but not limited to, diabetes mellitus, cardiovascular disease, cancer, and mental health. It is also important to recognise that these strategies may need to be tailored at a local level, given the wide disparities in the disease burden experienced by pregnant women across Indonesia and inequities in the access to care [34,35].

With the increasing rate of some NCDs and prevailing rate of infectious diseases among pregnant women in Indonesia, there is also the consideration of what implication this may have on maternal care, and consequently, maternal and neonatal outcomes and how Indonesia will achieve the 2030 SDG of a MMR of less than 70 [3]. In Indonesia, steady achievements have been made in improving access to maternal care with 91% of women delivering with a skilled birth attendant, including 74% as facility births, and over 77% of women being provided four or more antenatal care visits in the five years preceding the Indonesia Demographic and Health Survey in 2017. [36] Despite this, maternal health care in Indonesia still faces many challenges as highlighted in the recent Consolidated Report on Indonesia Health Sector Review 2018. These challenges include poor quality of care, ineffective referral systems, lack of compliance to basic protocol standards, as well as the lack of capacity of health centres and providers to treat complications, including high-risk pregnancies [33]. Only 70% of maternal healthcare facilities were reported to have the capacity to provide care for obstetric complications, such as haemorrhage, preeclampsia, and prolonged labour, which are classified as “direct” causes of maternal death.

The readiness of current maternal health services in primary care (public and private), the main provider accessed by pregnant women in Indonesia, is also a concern. Challenges include low availability of clinical guidelines and lack of provision of training for basic obstetric care, particularly in *Polindes* and *Pokedes* (village maternity posts and village health posts), and private maternal health providers [37]. There is also a lack of or no diagnostic capacity for some concomitant illnesses in primary care, particularly in private clinics and village maternity posts, such as for testing of haemoglobin, blood glucose, urine dipsticks for protein and glucose, and rapid testing kits for HIV and syphilis [37].

In light of these existing issues, this raises the concern of the Indonesian health system's preparedness to treat and manage the growing number of pregnancies with concomitant illnesses and what implications this may have. Of the limited literature in Indonesia focusing on maternal healthcare provision strategies for concomitant illnesses, common challenges highlighted included health providers being inadequately prepared to manage concomitant illnesses, along with gaps in clinical knowledge and skills, and a lack of use of practice guidelines in comparison to actual healthcare practices [38], [39], [40]. Challenges related to providing quality care to pregnant women with concomitant illnesses also included lack of infrastructure, access to appropriate diagnostic tools, and adequate support staff in public health centres [41], [42], [43]. However, training and education have shown to lead to improved maternal health outcomes for some concomitant illnesses in pregnancy in Indonesia [41].

From the perspective of financing, as Indonesia continues to roll out Universal Health Coverage (UHC), *Jamiman Kesehatan Nasional* (JKN), there may be implications of the national health insurance scheme to meet the demand to provide care for the rising burden of NCDs in pregnancy [44]. The World Bank highlighted this in a report assessing the supply-side readiness for UHC for NCDs in Indonesia. For example, the diagnostic and monitoring capability of *Puskesmas*, primary health care centres, for diabetes mellitus is a concern, with only 47% able to conduct urine tests and 54% able to conduct blood glucose tests [44]. This report also highlights the wide inequities in availability of care for diabetes mellitus across Indonesia [44]. For example, disparities are evident between rural and urban areas in Indonesia, with approximately a 20% less availability of diabetes diagnostic tests in rural areas [44]. Supply of diabetes care also varies between provinces, with less than 20% of *Puskesmas* in provinces such as Gorontalo, Papua, and Southeast Sulawesi having availability to provide such tests, in comparison to a 100% service coverage in Yogyakarta. [44].

How this can be addressed remains unclear. In LMICs, existing evidence has demonstrated the success of interventions focusing on ‘direct’ causes of maternal death, as well as some for concomitant illnesses in pregnancy, such as malaria and anaemia [27]. For example, for malaria in pregnancy key interventions include coordination of care with antenatal care for screening and provision of treatment, antimalarial medication, and provision of insecticide-treated nets. Despite progress made, Indonesia's approach to malaria in pregnancy still faces some challenges, such as limited resources [45], intervention coverage [46], dealing with multiple malaria strains[46], and a lack of policy adoption by the country to global WHO standards [47]. Existing interventions for some concomitant illness may provide lessons to address the growing burden of other concomitant illnesses. However, more evidence is needed on appropriate interventions and coordination of care for other concomitant illnesses, specifically NCDs, other infectious diseases, malnutrition, and mental health [27]. Potential avenues for Indonesia to address this problem is to strengthen integration of care between all existing health services and maternal care providers at all levels of care, both in the public and private sector, particularly for NCDs, as a part of Indonesia's national health systems strengthening plan for maternal health [33]. It may be beneficial to strengthen the screening and monitoring of NCDs in pregnancy by utilising existing community based maternal health programs, as well screening of NCDs by Integrated Health Posts, *Posyendu,* among pregnant women by integrating these existing infrastructures with maternal health providers. To meet the rising burden of NCDs as well as other infectious diseases in pregnancy, this may require an increase in funding by the *JKN*, such as increasing the minimum available visits to relevant providers or specialists for treatment and management of concomitant illnesses during pregnancy.

A strength of this study is that it utilised Sugarman and colleagues method for estimating tuberculosis in pregnant women in LMICs [6] and applied it to a range of illnesses for one country. As such, it allows for a more comprehensive picture of the burden of concomitant illnesses for pregnant women in Indonesia. This is a particularly useful method that could be adapted to other LMICs, where data availability on diseases in pregnancy may be limited. This study has some limitations. Firstly, the estimates derived in this study are subject to the limitations of the GBD methodology, which has been detailed elsewhere by the GBD Incidence and Prevalence Collaborators [48]. Secondly, for some concomitant illnesses in Indonesia incident data were not available, such as anemia, or cases were significantly low. Some concomitant illnesses were also grouped together with other conditions according to GBD code mapping methodology and did not allow for analysis of specific conditions such as hypertension. Thirdly, this study is reliant on the secondary data analysis of multiple sources therefore estimates may be overestimated or underestimated, particularly for incident estimates from the GBD. Source of data inputs are accessible from the GBD Data Input Sources Tool [49]. Fourthly, the future projections based on the model of this study are limited to extrapolation derived from existing estimates, which for some diseases there may be a small sample size or variability in the data. From 2020, it is not yet clear what impacts the COVID-19 global pandemic will have on pregnancy rates and outcomes. Lastly, we recognise that this study presents national estimates only and so does not identify regional variations in the burden of disease among Indonesian pregnant women such as across provinces, or between urban and rural areas. However, this study provides a comprehensive overview of national level epidemiological trends and future research could provide opportunities to conduct a comparative analysis of incident estimates of concomitant illnesses among pregnant women at these localised levels. Despite these limitations, these estimates of concomitant illnesses in pregnancy may provide guidance to stakeholders where practice, policy, and programmatic efforts for maternal healthcare in Indonesia could be focussed to so the health system may prepare to address both the persisting high rates of infectious diseases and increasing burden of NCDs in pregnancy.

## 5 Conclusion

This is the first time estimates across a wide range of concomitant illnesses in pregnant women has been undertaken for Indonesia. Given the rising burden of NCDs and persisting burden of infectious diseases estimated in pregnant women in Indonesia, more research is needed to understand how access and quality to care for Indonesian pregnant women with concomitant illnesses could be improved to reduce the potential impact on pregnancy outcomes, and for Indonesia to achieve the SDG of a MMR of less than 70 by 2030.

## Declaration of Competing Interest

We declare no competing interests.

## Appendix Supplementary materials

Image, application 1
