# Supplementary material for: Incidence of concomitant illnesses in pregnancy in Indonesia: Estimates from 1990–2019, with projections to 2030
Source: Lancet Reg Health West Pac. 2021 Apr 13;10:100139. doi: 10.1016/j.lanwpc.2021.100139 (PMC8315454; doi:10.1016/j.lanwpc.2021.100139)
Supplement: Supplementary file 1 [file mmc1.docx]

**Supplementary material**

**Table 1· Guidelines for Accurate and Transparent Health Estimates Reporting (GATHER) checklist of information that should be included in reports of global health estimates**

| **Item number** | **Checklist item** | **Section where reported** |
| --- | --- | --- |
| **Objectives and funding** | | |
| **1** | Define the indicator(s), populations (including age, sex, and geographic entities), and time period(s) for which estimates were made· | Methods (main text), Table 1, Table 2 |
| **2** | List the funding sources for the work· | Funding statement in manuscript |
| **Data inputs** | | |
| ***For all data inputs from multiple sources that are synthesised as part of the study*** | | |
| **3** | Describe how the data were identified and how the data were accessed· | Methods |
| **4** | Specify the inclusion and exclusion criteria· Identify all ad-hoc exclusions· | Methods |
| **5** | Provide information about all included data sources and their main characteristics· For each data source used, report reference information or contact name/institution, population represented, data collection method, year(s) of data collection, sex and age range, diagnostic criteria or measurement method, and sample size, as relevant· | Methods |
| **6** | Identify and describe any categories of input data that have potentially important biases (eg, based on characteristics listed in item 5)· | N/A |
| ***For data inputs that contribute to the analysis but were not synthesised as part of the study*** | | |
| **7** | Describe and give sources for any other data inputs· | Methods |
| **For all data inputs** | | |
| **9** | Provide a conceptual overview of the data analysis method· A diagram may be helpful· | Methods |
| **10** | Provide a detailed description of all steps of the analysis, including mathematical formulae· This description should cover, as relevant, data cleaning, data pre-processing, data adjustments and weighting of data sources, and mathematical or statistical model(s)· | Methods, Supplementary File |
| **11** | Describe how candidate models were evaluated and how the final model(s) were selected· | N/A |
| **12** | Provide the results of an evaluation of model performance, if done, as well as the results of any relevant sensitivity analysis· | N/A· |
| **13** | Describe methods of calculating uncertainty of the estimates· State which sources of uncertainty were, and were not, accounted for in the uncertainty analysis· | Method**s** |
| **14** | State how analytical or statistical source code used to generate estimates can be accessed· | Supplementary file.. |
| **Results and discussion** | | |
| **15** | Provide published estimates in a file format from which data can be efficiently extracted· | Results (main text), Table 1, Table 2, Supplementary file |
| **16** | Report a quantitative measure of the uncertainty of the estimates (eg, uncertainty intervals)· | Results (main text), Table 1, Table 2 |
| **17** | Interpret results in light of existing evidence·  If updating a previous set of estimates, describe the reasons for changes in estimates· | Discussion (main text) |
| **18** | Discuss limitations of the estimates· Include a discussion of any modelling assumptions or data limitations that affect interpretation of the estimates | Discussion (main text) |

**Statistical modelling used to estimate incidence of disease among pregnant women in Indonesia**

To determine estimates for observed and projected incidence of each disease of interest the following steps were followed. All analyses were performed using Stata v15.1 (StataCorp, College Station, TX).

**Step 1: Generate estimates for birth rate, population and incidence of disease**

A dataset was generated with 100 estimates for the crude birth rate, proportion of women aged 15-49 years and estimated incidence rate of disease for women aged 15-49 years based on the observed data for each year from 1990 to 2019.

The estimated incidence rate of disease for women aged 15 – 49 was determined by:

$$Incidence rate of disease in women \left( 15-49 years \right)=\frac{Incidence of disease in the population}{population of women \left( 15-49 years \right)} (1)$$

The 100 estimates were generated by first sampling from 1000 binomial distributions using the rbinomial(*n*, *p*) function in Stata^1^ with one trial (*n* = 1) and the observed proportion for the variable as the success probability (*p*). The proportion of ‘successes’ from these 1000 trials was then saved as the estimated proportion for the given year and this was repeated 100 times to generate 100 estimates for each of the three variables.

**Step 2: Generate estimates of cases of disease in pregnant women**

Using the method proposed by Sugarman et al^2^ we used the estimated incidence rate of disease in women aged 15-49 years to estimate the number of disease cases in pregnant women.

Firstly, we estimated the number of pregnant days within the country in a given year, based on the average gestational period of 280 days:

$$Estimated number of pregnant days =Total population \times crude birth rate \times\frac{280 days per pregnancy}{365 days per year} (2)$$

The estimated number of cases of disease in pregnant women within a year was then obtained by combining (1) and (2):

$$Number of cases of disease in pregnant women= Incidence rate of disease in women \times estimated number of pregnant days (3)$$

**Step 3: Extrapolate results to predict future estimates**

The data generated in Steps 1 and 2 were then used to produce a single estimate of the number of cases of disease in pregnant women for each year as well as extrapolating the results out to 2030. This required creating new empty observations for the projected years and then fitting a restricted cubic spline model3 to the data. We placed five knots at equally spaced percentiles as recommended by Harrell^4^. Predicted values were then extracted from the model for each year, including the additional years.

In addition to generating estimates for each year standard errors for each estimate were generated which were used to determine the 95% confidence interval for the predicted estimates.

These results were plotted to produce the Figures presented in the paper.

**Assumptions**

When extrapolating the data to the projected years the model assumed that population growth, pregnancy rates and incidence of the disease in pregnant women aged 15-49 years will follow similar trends to the observed data.

**References**

1. StataCorp. 2017. Stata Statistical Software: Release 15. College Station, TX: StataCorp LLC.
2. Sugarman J, Colvin C, Moran AC, Oxlade O. Tuberculosis in pregnancy: an estimate of the global burden of disease. Lancet Glob Health 2014; 2.
3. Harrell FE Jr, Lee KL, Pollock BG. Regression models in clinical studies: determining relationships between predictors and response. J Natl Cancer Inst. 1988 Oct 5;80(15):1198-202. doi: 10.1093/jnci/80.15.1198. PMID: 3047407.
4. Harrell FE, Jr. Regression Modelling Strategies: With Applications to Linear Models, Logistic Regression, and Survival Analysis. New York: Springer; 2001.

**Tables: Estimated incidence of disease in pregnant women in Indonesia, 1990-2030**

Note: Future trends from 2020 to 2030 were projected according to statistical modelling methods described in the Supplementary Material under “Step 3: Extrapolate results to predict future estimates”.

| **Acute hepatitis** | | | | |
| --- | --- | --- | --- | --- |
| **Year** | **Mean** | **Standard Error of Mean** | **Min** | **Max** |
| 1990 | 108308 | 1858 | 104666 | 111951 |
| 1991 | 107064 | 1508 | 104109 | 110019 |
| 1992 | 105831 | 1214 | 103450 | 108211 |
| 1993 | 104661 | 1025 | 102652 | 106670 |
| 1994 | 103619 | 971 | 101715 | 105522 |
| 1995 | 102768 | 1024 | 100762 | 104774 |
| 1996 | 102173 | 1111 | 99996 | 104349 |
| 1997 | 101896 | 1170 | 99603 | 104190 |
| 1998 | 102003 | 1166 | 99717 | 104288 |
| 1999 | 102525 | 1095 | 100379 | 104671 |
| 2000 | 103373 | 1007 | 101401 | 105346 |
| 2001 | 104427 | 964 | 102536 | 106317 |
| 2002 | 105564 | 1003 | 103597 | 107530 |
| 2003 | 106665 | 1097 | 104514 | 108816 |
| 2004 | 107608 | 1186 | 105284 | 109932 |
| 2005 | 108272 | 1210 | 105900 | 110644 |
| 2006 | 108560 | 1146 | 106313 | 110807 |
| 2007 | 108459 | 1043 | 106414 | 110504 |
| 2008 | 107981 | 978 | 106064 | 109897 |
| 2009 | 107135 | 1001 | 105173 | 109098 |
| 2010 | 105934 | 1092 | 103795 | 108074 |
| 2011 | 104388 | 1177 | 102083 | 106694 |
| 2012 | 102515 | 1193 | 100177 | 104852 |
| 2013 | 100354 | 1138 | 98125 | 102584 |
| 2014 | 97955 | 1047 | 95904 | 100007 |
| 2015 | 95365 | 984 | 93437 | 97294 |
| 2016 | 92632 | 1027 | 90619 | 94646 |
| 2017 | 89804 | 1215 | 87423 | 92184 |
| 2018 | 86927 | 1514 | 83960 | 89895 |
| 2019 | 84043 | 1875 | 80368 | 87718 |
| 2020 | 81159 | 2268 | 76714 | 85604 |
| 2021 | 78275 | 2679 | 73024 | 83525 |
| 2022 | 75391 | 3101 | 69314 | 81467 |
| 2023 | 72506 | 3529 | 65589 | 79423 |
| 2024 | 69622 | 3962 | 61856 | 77388 |
| 2025 | 66738 | 4399 | 58116 | 75359 |
| 2026 | 63854 | 4838 | 54372 | 73335 |
| 2027 | 60969 | 5278 | 50624 | 71315 |
| 2028 | 58085 | 5720 | 46873 | 69297 |
| 2029 | 55201 | 6164 | 43120 | 67281 |
| 2030 | 52317 | 6608 | 39366 | 65267 |

| **Anxiety** | | | | |
| --- | --- | --- | --- | --- |
| **Year** | **Mean** | **Standard Error of Mean** | **Min** | **Max** |
| 1990 | 31866 | 836 | 30228 | 33505 |
| 1991 | 31487 | 678 | 30157 | 32816 |
| 1992 | 31110 | 546 | 30039 | 32181 |
| 1993 | 30752 | 461 | 29848 | 31656 |
| 1994 | 30431 | 437 | 29574 | 31287 |
| 1995 | 30164 | 460 | 29262 | 31067 |
| 1996 | 29971 | 500 | 28991 | 30950 |
| 1997 | 29869 | 526 | 28837 | 30901 |
| 1998 | 29877 | 525 | 28848 | 30905 |
| 1999 | 30006 | 493 | 29040 | 30971 |
| 2000 | 30243 | 453 | 29355 | 31130 |
| 2001 | 30566 | 434 | 29716 | 31417 |
| 2002 | 30956 | 451 | 30072 | 31841 |
| 2003 | 31391 | 494 | 30424 | 32359 |
| 2004 | 31852 | 533 | 30806 | 32897 |
| 2005 | 32316 | 544 | 31249 | 33383 |
| 2006 | 32764 | 516 | 31753 | 33774 |
| 2007 | 33172 | 469 | 32252 | 34092 |
| 2008 | 33520 | 440 | 32658 | 34382 |
| 2009 | 33783 | 450 | 32901 | 34666 |
| 2010 | 33941 | 491 | 32978 | 34903 |
| 2011 | 33969 | 529 | 32932 | 35007 |
| 2012 | 33854 | 537 | 32803 | 34906 |
| 2013 | 33609 | 512 | 32606 | 34612 |
| 2014 | 33257 | 471 | 32334 | 34179 |
| 2015 | 32817 | 443 | 31950 | 33685 |
| 2016 | 32313 | 462 | 31407 | 33219 |
| 2017 | 31766 | 546 | 30695 | 32837 |
| 2018 | 31197 | 681 | 29862 | 32532 |
| 2019 | 30625 | 844 | 28972 | 32278 |
| 2020 | 30053 | 1020 | 28053 | 32052 |
| 2021 | 29480 | 1205 | 27118 | 31842 |
| 2022 | 28908 | 1395 | 26174 | 31642 |
| 2023 | 28335 | 1588 | 25224 | 31447 |
| 2024 | 27763 | 1783 | 24269 | 31257 |
| 2025 | 27191 | 1979 | 23312 | 31069 |
| 2026 | 26618 | 2176 | 22353 | 30884 |
| 2027 | 26046 | 2375 | 21392 | 30700 |
| 2028 | 25473 | 2573 | 20429 | 30517 |
| 2029 | 24901 | 2773 | 19466 | 30336 |
| 2030 | 24329 | 2973 | 18502 | 30155 |

| **Bipolar disorder** | | | | |
| --- | --- | --- | --- | --- |
| **Year** | **Mean** | **Standard Error of Mean** | **Min** | **Max** |
| 1990 | 1472 | 156 | 1167 | 1776 |
| 1991 | 1484 | 126 | 1237 | 1732 |
| 1992 | 1497 | 102 | 1298 | 1696 |
| 1993 | 1509 | 86 | 1341 | 1678 |
| 1994 | 1522 | 81 | 1362 | 1681 |
| 1995 | 1534 | 86 | 1366 | 1702 |
| 1996 | 1545 | 93 | 1363 | 1727 |
| 1997 | 1556 | 98 | 1364 | 1748 |
| 1998 | 1566 | 98 | 1374 | 1757 |
| 1999 | 1575 | 92 | 1395 | 1754 |
| 2000 | 1583 | 84 | 1418 | 1748 |
| 2001 | 1590 | 81 | 1432 | 1748 |
| 2002 | 1597 | 84 | 1432 | 1761 |
| 2003 | 1603 | 92 | 1423 | 1783 |
| 2004 | 1608 | 99 | 1413 | 1802 |
| 2005 | 1612 | 101 | 1414 | 1811 |
| 2006 | 1616 | 96 | 1428 | 1804 |
| 2007 | 1618 | 87 | 1447 | 1789 |
| 2008 | 1616 | 82 | 1455 | 1776 |
| 2009 | 1609 | 84 | 1445 | 1773 |
| 2010 | 1595 | 91 | 1416 | 1774 |
| 2011 | 1574 | 98 | 1381 | 1767 |
| 2012 | 1543 | 100 | 1347 | 1739 |
| 2013 | 1504 | 95 | 1318 | 1691 |
| 2014 | 1459 | 88 | 1287 | 1631 |
| 2015 | 1408 | 82 | 1246 | 1569 |
| 2016 | 1353 | 86 | 1184 | 1521 |
| 2017 | 1295 | 102 | 1096 | 1494 |
| 2018 | 1236 | 127 | 987 | 1484 |
| 2019 | 1177 | 157 | 869 | 1484 |
| 2020 | 1117 | 190 | 745 | 1489 |
| 2021 | 1058 | 224 | 618 | 1497 |
| 2022 | 998 | 260 | 490 | 1507 |
| 2023 | 939 | 295 | 360 | 1518 |
| 2024 | 880 | 332 | 230 | 1530 |
| 2025 | 820 | 368 | 99 | 1542 |
| 2026 | 761 | 405 | -33 | 1555 |
| 2027 | 702 | 442 | -164 | 1568 |
| 2028 | 642 | 479 | -296 | 1581 |
| 2029 | 583 | 516 | -428 | 1594 |
| 2030 | 524 | 553 | -561 | 1608 |

| **Brain and nervous system cancer** | | | | |
| --- | --- | --- | --- | --- |
| **Year** | **Mean** | **Standard Error of Mean** | **Min** | **Max** |
| 1990 | 11 | 26 | -41 | 62 |
| 1991 | 21 | 21 | -21 | 62 |
| 1992 | 30 | 17 | -3 | 64 |
| 1993 | 40 | 14 | 11 | 68 |
| 1994 | 48 | 14 | 22 | 75 |
| 1995 | 56 | 14 | 28 | 84 |
| 1996 | 62 | 16 | 31 | 93 |
| 1997 | 66 | 16 | 34 | 98 |
| 1998 | 68 | 16 | 36 | 100 |
| 1999 | 68 | 15 | 38 | 98 |
| 2000 | 65 | 14 | 38 | 93 |
| 2001 | 61 | 14 | 35 | 88 |
| 2002 | 56 | 14 | 29 | 84 |
| 2003 | 51 | 15 | 21 | 81 |
| 2004 | 45 | 17 | 13 | 78 |
| 2005 | 41 | 17 | 7 | 74 |
| 2006 | 37 | 16 | 5 | 68 |
| 2007 | 34 | 15 | 5 | 63 |
| 2008 | 32 | 14 | 5 | 59 |
| 2009 | 31 | 14 | 4 | 59 |
| 2010 | 32 | 15 | 1 | 62 |
| 2011 | 33 | 17 | 0 | 65 |
| 2012 | 35 | 17 | 2 | 68 |
| 2013 | 37 | 16 | 6 | 69 |
| 2014 | 41 | 15 | 12 | 70 |
| 2015 | 45 | 14 | 18 | 72 |
| 2016 | 49 | 14 | 21 | 77 |
| 2017 | 53 | 17 | 20 | 87 |
| 2018 | 58 | 21 | 16 | 100 |
| 2019 | 63 | 26 | 11 | 115 |
| 2020 | 67 | 32 | 5 | 130 |
| 2021 | 72 | 38 | -2 | 146 |
| 2022 | 77 | 44 | -9 | 162 |
| 2023 | 81 | 50 | -16 | 179 |
| 2024 | 86 | 56 | -23 | 195 |
| 2025 | 91 | 62 | -31 | 212 |
| 2026 | 95 | 68 | -38 | 229 |
| 2027 | 100 | 74 | -46 | 246 |
| 2028 | 105 | 81 | -53 | 263 |
| 2029 | 109 | 87 | -61 | 280 |
| 2030 | 114 | 93 | -68 | 297 |

| **Breast cancer** | | | | |
| --- | --- | --- | --- | --- |
| **Year** | **Mean** | **Standard Error of Mean** | **Min** | **Max** |
| 1990 | 806 | 133 | 546 | 1067 |
| 1991 | 814 | 108 | 603 | 1025 |
| 1992 | 821 | 87 | 651 | 991 |
| 1993 | 829 | 73 | 686 | 973 |
| 1994 | 839 | 69 | 703 | 975 |
| 1995 | 850 | 73 | 707 | 994 |
| 1996 | 865 | 79 | 709 | 1020 |
| 1997 | 883 | 84 | 719 | 1046 |
| 1998 | 904 | 83 | 741 | 1068 |
| 1999 | 931 | 78 | 778 | 1084 |
| 2000 | 962 | 72 | 821 | 1103 |
| 2001 | 997 | 69 | 862 | 1132 |
| 2002 | 1037 | 72 | 896 | 1177 |
| 2003 | 1080 | 78 | 926 | 1233 |
| 2004 | 1126 | 85 | 960 | 1292 |
| 2005 | 1175 | 86 | 1006 | 1344 |
| 2006 | 1227 | 82 | 1066 | 1387 |
| 2007 | 1277 | 75 | 1131 | 1423 |
| 2008 | 1324 | 70 | 1187 | 1461 |
| 2009 | 1362 | 72 | 1222 | 1502 |
| 2010 | 1389 | 78 | 1236 | 1542 |
| 2011 | 1401 | 84 | 1236 | 1566 |
| 2012 | 1396 | 85 | 1229 | 1563 |
| 2013 | 1374 | 81 | 1215 | 1533 |
| 2014 | 1339 | 75 | 1193 | 1486 |
| 2015 | 1294 | 70 | 1156 | 1432 |
| 2016 | 1241 | 73 | 1097 | 1384 |
| 2017 | 1182 | 87 | 1012 | 1352 |
| 2018 | 1121 | 108 | 909 | 1333 |
| 2019 | 1059 | 134 | 796 | 1321 |
| 2020 | 997 | 162 | 679 | 1315 |
| 2021 | 935 | 191 | 560 | 1310 |
| 2022 | 874 | 221 | 439 | 1308 |
| 2023 | 812 | 252 | 318 | 1306 |
| 2024 | 750 | 283 | 195 | 1305 |
| 2025 | 688 | 314 | 72 | 1304 |
| 2026 | 627 | 346 | -51 | 1304 |
| 2027 | 565 | 377 | -174 | 1304 |
| 2028 | 503 | 409 | -298 | 1304 |
| 2029 | 441 | 440 | -422 | 1304 |
| 2030 | 380 | 472 | -546 | 1305 |

| **Cardiomyopathy and myocarditis** | | | | |
| --- | --- | --- | --- | --- |
| **Year** | **Mean** | **Standard Error of Mean** | **Min** | **Max** |
| 1990 | 345 | 73 | 202 | 488 |
| 1991 | 339 | 59 | 223 | 455 |
| 1992 | 333 | 48 | 239 | 426 |
| 1993 | 327 | 40 | 248 | 406 |
| 1994 | 322 | 38 | 247 | 397 |
| 1995 | 319 | 40 | 240 | 398 |
| 1996 | 318 | 44 | 232 | 403 |
| 1997 | 319 | 46 | 229 | 409 |
| 1998 | 324 | 46 | 234 | 414 |
| 1999 | 332 | 43 | 247 | 416 |
| 2000 | 342 | 40 | 264 | 419 |
| 2001 | 353 | 38 | 279 | 427 |
| 2002 | 364 | 39 | 287 | 442 |
| 2003 | 374 | 43 | 290 | 459 |
| 2004 | 382 | 47 | 290 | 473 |
| 2005 | 385 | 48 | 292 | 479 |
| 2006 | 384 | 45 | 296 | 473 |
| 2007 | 380 | 41 | 299 | 460 |
| 2008 | 372 | 38 | 296 | 447 |
| 2009 | 362 | 39 | 285 | 439 |
| 2010 | 351 | 43 | 267 | 435 |
| 2011 | 341 | 46 | 250 | 432 |
| 2012 | 331 | 47 | 240 | 423 |
| 2013 | 323 | 45 | 235 | 411 |
| 2014 | 315 | 41 | 235 | 396 |
| 2015 | 308 | 39 | 232 | 384 |
| 2016 | 302 | 40 | 223 | 381 |
| 2017 | 295 | 48 | 202 | 389 |
| 2018 | 289 | 59 | 173 | 406 |
| 2019 | 283 | 74 | 139 | 428 |
| 2020 | 277 | 89 | 103 | 452 |
| 2021 | 271 | 105 | 65 | 478 |
| 2022 | 265 | 122 | 27 | 504 |
| 2023 | 259 | 139 | -12 | 531 |
| 2024 | 253 | 156 | -52 | 558 |
| 2025 | 247 | 173 | -91 | 586 |
| 2026 | 241 | 190 | -131 | 614 |
| 2027 | 235 | 207 | -171 | 642 |
| 2028 | 229 | 225 | -211 | 670 |
| 2029 | 223 | 242 | -251 | 698 |
| 2030 | 217 | 260 | -291 | 726 |

| **Cervical cancer** | | | | |
| --- | --- | --- | --- | --- |
| **Year** | **Mean** | **Standard Error of Mean** | **Min** | **Max** |
| 1990 | 434 | 84 | 270 | 598 |
| 1991 | 432 | 68 | 299 | 565 |
| 1992 | 430 | 55 | 323 | 537 |
| 1993 | 429 | 46 | 338 | 519 |
| 1994 | 428 | 44 | 343 | 514 |
| 1995 | 429 | 46 | 339 | 519 |
| 1996 | 432 | 50 | 335 | 530 |
| 1997 | 438 | 53 | 335 | 541 |
| 1998 | 447 | 52 | 345 | 550 |
| 1999 | 460 | 49 | 363 | 556 |
| 2000 | 474 | 45 | 386 | 563 |
| 2001 | 490 | 43 | 405 | 575 |
| 2002 | 504 | 45 | 416 | 593 |
| 2003 | 517 | 49 | 420 | 613 |
| 2004 | 525 | 53 | 421 | 630 |
| 2005 | 529 | 54 | 422 | 635 |
| 2006 | 526 | 52 | 425 | 627 |
| 2007 | 517 | 47 | 426 | 609 |
| 2008 | 505 | 44 | 419 | 591 |
| 2009 | 489 | 45 | 401 | 577 |
| 2010 | 471 | 49 | 375 | 567 |
| 2011 | 453 | 53 | 349 | 556 |
| 2012 | 434 | 54 | 329 | 539 |
| 2013 | 416 | 51 | 316 | 517 |
| 2014 | 399 | 47 | 307 | 491 |
| 2015 | 381 | 44 | 295 | 468 |
| 2016 | 364 | 46 | 274 | 455 |
| 2017 | 347 | 55 | 240 | 454 |
| 2018 | 330 | 68 | 196 | 463 |
| 2019 | 313 | 84 | 148 | 478 |
| 2020 | 296 | 102 | 96 | 495 |
| 2021 | 279 | 120 | 43 | 515 |
| 2022 | 262 | 139 | -12 | 535 |
| 2023 | 244 | 159 | -66 | 555 |
| 2024 | 227 | 178 | -122 | 576 |
| 2025 | 210 | 198 | -177 | 598 |
| 2026 | 193 | 217 | -233 | 619 |
| 2027 | 176 | 237 | -289 | 641 |
| 2028 | 159 | 257 | -345 | 663 |
| 2029 | 142 | 277 | -401 | 685 |
| 2030 | 125 | 297 | -457 | 707 |

| **Chronic kidney disease** | | | | |
| --- | --- | --- | --- | --- |
| **Year** | **Mean** | **Standard Error of Mean** | **Min** | **Max** |
| 1990 | 1510 | 182 | 1153 | 1868 |
| 1991 | 1542 | 148 | 1252 | 1832 |
| 1992 | 1574 | 119 | 1340 | 1807 |
| 1993 | 1605 | 101 | 1408 | 1802 |
| 1994 | 1637 | 95 | 1450 | 1823 |
| 1995 | 1668 | 100 | 1471 | 1864 |
| 1996 | 1698 | 109 | 1485 | 1912 |
| 1997 | 1729 | 115 | 1504 | 1954 |
| 1998 | 1758 | 114 | 1534 | 1983 |
| 1999 | 1788 | 107 | 1577 | 1998 |
| 2000 | 1818 | 99 | 1625 | 2012 |
| 2001 | 1851 | 95 | 1666 | 2037 |
| 2002 | 1889 | 98 | 1696 | 2082 |
| 2003 | 1932 | 108 | 1721 | 2143 |
| 2004 | 1984 | 116 | 1756 | 2212 |
| 2005 | 2044 | 119 | 1812 | 2277 |
| 2006 | 2115 | 112 | 1894 | 2335 |
| 2007 | 2191 | 102 | 1991 | 2392 |
| 2008 | 2268 | 96 | 2080 | 2456 |
| 2009 | 2341 | 98 | 2148 | 2533 |
| 2010 | 2403 | 107 | 2193 | 2613 |
| 2011 | 2451 | 115 | 2224 | 2677 |
| 2012 | 2479 | 117 | 2250 | 2709 |
| 2013 | 2490 | 112 | 2272 | 2709 |
| 2014 | 2487 | 103 | 2286 | 2688 |
| 2015 | 2472 | 97 | 2283 | 2661 |
| 2016 | 2449 | 101 | 2251 | 2646 |
| 2017 | 2419 | 119 | 2186 | 2653 |
| 2018 | 2387 | 149 | 2096 | 2678 |
| 2019 | 2354 | 184 | 1994 | 2715 |
| 2020 | 2322 | 222 | 1886 | 2758 |
| 2021 | 2289 | 263 | 1774 | 2804 |
| 2022 | 2256 | 304 | 1660 | 2852 |
| 2023 | 2224 | 346 | 1545 | 2902 |
| 2024 | 2191 | 389 | 1429 | 2953 |
| 2025 | 2158 | 431 | 1312 | 3004 |
| 2026 | 2125 | 475 | 1195 | 3055 |
| 2027 | 2093 | 518 | 1078 | 3107 |
| 2028 | 2060 | 561 | 960 | 3160 |
| 2029 | 2027 | 605 | 842 | 3212 |
| 2030 | 1994 | 648 | 724 | 3265 |

| **Colon rectum cancer** | | | | |
| --- | --- | --- | --- | --- |
| **Year** | **Mean** | **Standard Error of Mean** | **Min** | **Max** |
| 1990 | 82 | 42 | 0 | 165 |
| 1991 | 87 | 34 | 21 | 154 |
| 1992 | 92 | 27 | 39 | 146 |
| 1993 | 97 | 23 | 52 | 142 |
| 1994 | 101 | 22 | 58 | 144 |
| 1995 | 105 | 23 | 60 | 150 |
| 1996 | 107 | 25 | 58 | 157 |
| 1997 | 109 | 26 | 57 | 161 |
| 1998 | 109 | 26 | 57 | 160 |
| 1999 | 107 | 25 | 59 | 156 |
| 2000 | 105 | 23 | 60 | 149 |
| 2001 | 102 | 22 | 59 | 144 |
| 2002 | 99 | 23 | 55 | 143 |
| 2003 | 98 | 25 | 49 | 146 |
| 2004 | 98 | 27 | 46 | 150 |
| 2005 | 101 | 27 | 47 | 154 |
| 2006 | 106 | 26 | 55 | 157 |
| 2007 | 113 | 24 | 67 | 160 |
| 2008 | 122 | 22 | 79 | 165 |
| 2009 | 130 | 23 | 86 | 174 |
| 2010 | 137 | 25 | 88 | 185 |
| 2011 | 141 | 27 | 89 | 193 |
| 2012 | 142 | 27 | 89 | 195 |
| 2013 | 140 | 26 | 90 | 190 |
| 2014 | 136 | 24 | 89 | 182 |
| 2015 | 129 | 22 | 86 | 173 |
| 2016 | 121 | 23 | 76 | 167 |
| 2017 | 112 | 27 | 59 | 166 |
| 2018 | 103 | 34 | 36 | 170 |
| 2019 | 93 | 42 | 10 | 176 |
| 2020 | 84 | 51 | -17 | 184 |
| 2021 | 74 | 60 | -44 | 192 |
| 2022 | 64 | 70 | -73 | 201 |
| 2023 | 55 | 80 | -101 | 211 |
| 2024 | 45 | 89 | -130 | 220 |
| 2025 | 36 | 99 | -159 | 230 |
| 2026 | 26 | 109 | -188 | 240 |
| 2027 | 16 | 119 | -217 | 250 |
| 2028 | 7 | 129 | -246 | 260 |
| 2029 | -3 | 139 | -275 | 270 |
| 2030 | -12 | 149 | -304 | 280 |

| **Chronic obstructive pulmonary disease** | | | | |
| --- | --- | --- | --- | --- |
| **Year** | **Mean** | **Standard Error of Mean** | **Min** | **Max** |
| 1990 | 2085 | 192 | 1709 | 2461 |
| 1991 | 2095 | 156 | 1791 | 2400 |
| 1992 | 2106 | 125 | 1860 | 2352 |
| 1993 | 2117 | 106 | 1910 | 2324 |
| 1994 | 2129 | 100 | 1933 | 2326 |
| 1995 | 2143 | 106 | 1936 | 2350 |
| 1996 | 2159 | 115 | 1935 | 2384 |
| 1997 | 2178 | 121 | 1941 | 2415 |
| 1998 | 2200 | 120 | 1964 | 2436 |
| 1999 | 2225 | 113 | 2004 | 2447 |
| 2000 | 2254 | 104 | 2050 | 2457 |
| 2001 | 2285 | 99 | 2090 | 2480 |
| 2002 | 2317 | 104 | 2114 | 2519 |
| 2003 | 2349 | 113 | 2127 | 2571 |
| 2004 | 2382 | 122 | 2142 | 2621 |
| 2005 | 2413 | 125 | 2168 | 2658 |
| 2006 | 2442 | 118 | 2210 | 2674 |
| 2007 | 2468 | 108 | 2257 | 2679 |
| 2008 | 2487 | 101 | 2289 | 2685 |
| 2009 | 2499 | 103 | 2296 | 2701 |
| 2010 | 2500 | 113 | 2280 | 2721 |
| 2011 | 2490 | 121 | 2252 | 2728 |
| 2012 | 2466 | 123 | 2225 | 2707 |
| 2013 | 2431 | 117 | 2201 | 2661 |
| 2014 | 2385 | 108 | 2173 | 2597 |
| 2015 | 2331 | 101 | 2132 | 2530 |
| 2016 | 2272 | 106 | 2064 | 2479 |
| 2017 | 2208 | 125 | 1962 | 2453 |
| 2018 | 2142 | 156 | 1836 | 2448 |
| 2019 | 2076 | 193 | 1697 | 2455 |
| 2020 | 2010 | 234 | 1551 | 2469 |
| 2021 | 1944 | 276 | 1402 | 2486 |
| 2022 | 1878 | 320 | 1251 | 2505 |
| 2023 | 1812 | 364 | 1098 | 2525 |
| 2024 | 1746 | 409 | 945 | 2547 |
| 2025 | 1680 | 454 | 790 | 2569 |
| 2026 | 1614 | 499 | 636 | 2592 |
| 2027 | 1548 | 545 | 480 | 2615 |
| 2028 | 1482 | 590 | 325 | 2638 |
| 2029 | 1416 | 636 | 169 | 2662 |
| 2030 | 1349 | 682 | 13 | 2685 |

| **Dengue** | | | | |
| --- | --- | --- | --- | --- |
| **Year** | **Mean** | **Standard Error of Mean** | **Min** | **Max** |
| 1990 | 45756 | 964 | 43867 | 47645 |
| 1991 | 43932 | 782 | 42399 | 45464 |
| 1992 | 42120 | 630 | 40885 | 43355 |
| 1993 | 40383 | 532 | 39341 | 41425 |
| 1994 | 38796 | 504 | 37809 | 39783 |
| 1995 | 37433 | 531 | 36393 | 38474 |
| 1996 | 36370 | 576 | 35241 | 37499 |
| 1997 | 35680 | 607 | 34491 | 36870 |
| 1998 | 35440 | 605 | 34254 | 36625 |
| 1999 | 35691 | 568 | 34578 | 36804 |
| 2000 | 36350 | 522 | 35327 | 37373 |
| 2001 | 37300 | 500 | 36320 | 38281 |
| 2002 | 38426 | 520 | 37406 | 39445 |
| 2003 | 39610 | 569 | 38494 | 40725 |
| 2004 | 40736 | 615 | 39531 | 41942 |
| 2005 | 41690 | 628 | 40459 | 42920 |
| 2006 | 42377 | 595 | 41212 | 43542 |
| 2007 | 42803 | 541 | 41742 | 43864 |
| 2008 | 42996 | 507 | 42002 | 43990 |
| 2009 | 42983 | 519 | 41965 | 44001 |
| 2010 | 42794 | 566 | 41684 | 43904 |
| 2011 | 42456 | 610 | 41260 | 43652 |
| 2012 | 41996 | 619 | 40783 | 43208 |
| 2013 | 41432 | 590 | 40276 | 42589 |
| 2014 | 40783 | 543 | 39719 | 41847 |
| 2015 | 40065 | 510 | 39065 | 41065 |
| 2016 | 39295 | 533 | 38251 | 40340 |
| 2017 | 38491 | 630 | 37257 | 39726 |
| 2018 | 37670 | 785 | 36131 | 39209 |
| 2019 | 36846 | 973 | 34940 | 38752 |
| 2020 | 36022 | 1176 | 33716 | 38328 |
| 2021 | 35198 | 1389 | 32474 | 37921 |
| 2022 | 34374 | 1608 | 31222 | 37526 |
| 2023 | 33550 | 1830 | 29962 | 37137 |
| 2024 | 32725 | 2055 | 28697 | 36753 |
| 2025 | 31901 | 2282 | 27430 | 36373 |
| 2026 | 31077 | 2509 | 26159 | 35995 |
| 2027 | 30253 | 2738 | 24887 | 35619 |
| 2028 | 29429 | 2967 | 23614 | 35244 |
| 2029 | 28605 | 3197 | 22339 | 34871 |
| 2030 | 27781 | 3427 | 21064 | 34498 |

| **Depressive disorders** | | | | |
| --- | --- | --- | --- | --- |
| **Year** | **Mean** | **Standard Error of Mean** | **Min** | **Max** |
| 1990 | 100161 | 1779 | 96674 | 103648 |
| 1991 | 98189 | 1443 | 95360 | 101018 |
| 1992 | 96234 | 1163 | 93955 | 98512 |
| 1993 | 94377 | 981 | 92454 | 96300 |
| 1994 | 92717 | 930 | 90894 | 94539 |
| 1995 | 91351 | 980 | 89431 | 93272 |
| 1996 | 90380 | 1063 | 88296 | 92463 |
| 1997 | 89899 | 1120 | 87704 | 92095 |
| 1998 | 90009 | 1116 | 87822 | 92197 |
| 1999 | 90762 | 1048 | 88707 | 92816 |
| 2000 | 92023 | 963 | 90135 | 93911 |
| 2001 | 93614 | 923 | 91804 | 95423 |
| 2002 | 95355 | 960 | 93473 | 97238 |
| 2003 | 97067 | 1051 | 95008 | 99127 |
| 2004 | 98571 | 1135 | 96347 | 100796 |
| 2005 | 99687 | 1159 | 97417 | 101958 |
| 2006 | 100283 | 1097 | 98133 | 102434 |
| 2007 | 100417 | 999 | 98459 | 102374 |
| 2008 | 100191 | 936 | 98357 | 102026 |
| 2009 | 99711 | 958 | 97833 | 101590 |
| 2010 | 99082 | 1045 | 97034 | 101130 |
| 2011 | 98406 | 1126 | 96199 | 100614 |
| 2012 | 97771 | 1142 | 95533 | 100008 |
| 2013 | 97185 | 1089 | 95051 | 99319 |
| 2014 | 96641 | 1002 | 94678 | 98605 |
| 2015 | 96131 | 942 | 94285 | 97977 |
| 2016 | 95646 | 984 | 93719 | 97574 |
| 2017 | 95178 | 1163 | 92899 | 97457 |
| 2018 | 94718 | 1449 | 91877 | 97558 |
| 2019 | 94259 | 1795 | 90741 | 97777 |
| 2020 | 93801 | 2171 | 89546 | 98056 |
| 2021 | 93342 | 2564 | 88316 | 98368 |
| 2022 | 92884 | 2968 | 87067 | 98701 |
| 2023 | 92425 | 3378 | 85804 | 99046 |
| 2024 | 91967 | 3793 | 84533 | 99401 |
| 2025 | 91508 | 4211 | 83255 | 99761 |
| 2026 | 91050 | 4631 | 81973 | 100126 |
| 2027 | 90591 | 5053 | 80688 | 100494 |
| 2028 | 90132 | 5476 | 79400 | 100865 |
| 2029 | 89674 | 5900 | 78110 | 101238 |
| 2030 | 89215 | 6325 | 76818 | 101612 |

| **Diabetes mellitus** | | | | |
| --- | --- | --- | --- | --- |
| **Year** | **Mean** | **Standard Error of Mean** | **Min** | **Max** |
| 1990 | 3610 | 297 | 3027 | 4193 |
| 1991 | 3928 | 241 | 3455 | 4401 |
| 1992 | 4244 | 194 | 3863 | 4625 |
| 1993 | 4547 | 164 | 4226 | 4869 |
| 1994 | 4824 | 155 | 4520 | 5129 |
| 1995 | 5063 | 164 | 4741 | 5384 |
| 1996 | 5249 | 178 | 4900 | 5597 |
| 1997 | 5370 | 187 | 5003 | 5738 |
| 1998 | 5414 | 187 | 5048 | 5780 |
| 1999 | 5373 | 175 | 5030 | 5717 |
| 2000 | 5264 | 161 | 4948 | 5580 |
| 2001 | 5108 | 154 | 4806 | 5411 |
| 2002 | 4929 | 161 | 4614 | 5244 |
| 2003 | 4748 | 176 | 4403 | 5092 |
| 2004 | 4587 | 190 | 4215 | 4959 |
| 2005 | 4470 | 194 | 4090 | 4849 |
| 2006 | 4414 | 183 | 4054 | 4773 |
| 2007 | 4422 | 167 | 4095 | 4749 |
| 2008 | 4494 | 157 | 4187 | 4801 |
| 2009 | 4628 | 160 | 4314 | 4942 |
| 2010 | 4825 | 175 | 4482 | 5167 |
| 2011 | 5082 | 188 | 4713 | 5451 |
| 2012 | 5398 | 191 | 5024 | 5772 |
| 2013 | 5765 | 182 | 5409 | 6122 |
| 2014 | 6176 | 168 | 5848 | 6504 |
| 2015 | 6621 | 157 | 6312 | 6930 |
| 2016 | 7092 | 164 | 6769 | 7414 |
| 2017 | 7579 | 194 | 7198 | 7960 |
| 2018 | 8076 | 242 | 7601 | 8551 |
| 2019 | 8574 | 300 | 7985 | 9162 |
| 2020 | 9071 | 363 | 8360 | 9783 |
| 2021 | 9569 | 429 | 8729 | 10410 |
| 2022 | 10067 | 496 | 9094 | 11040 |
| 2023 | 10565 | 565 | 9458 | 11672 |
| 2024 | 11063 | 634 | 9820 | 12306 |
| 2025 | 11561 | 704 | 10181 | 12941 |
| 2026 | 12058 | 774 | 10541 | 13576 |
| 2027 | 12556 | 845 | 10900 | 14212 |
| 2028 | 13054 | 916 | 11260 | 14849 |
| 2029 | 13552 | 987 | 11618 | 15486 |
| 2030 | 14050 | 1058 | 11977 | 16123 |

| **HIV/AIDs** | | | | |
| --- | --- | --- | --- | --- |
| **Year** | **Mean** | **Standard Error of Mean** | **Min** | **Max** |
| 1990 | 13 | 46 | -78 | 104 |
| 1991 | 20 | 38 | -54 | 94 |
| 1992 | 27 | 30 | -32 | 87 |
| 1993 | 35 | 26 | -15 | 85 |
| 1994 | 42 | 24 | -6 | 89 |
| 1995 | 49 | 26 | -1 | 99 |
| 1996 | 56 | 28 | 2 | 111 |
| 1997 | 64 | 29 | 6 | 121 |
| 1998 | 71 | 29 | 14 | 128 |
| 1999 | 78 | 27 | 24 | 131 |
| 2000 | 85 | 25 | 36 | 134 |
| 2001 | 94 | 24 | 46 | 141 |
| 2002 | 103 | 25 | 54 | 152 |
| 2003 | 114 | 27 | 61 | 168 |
| 2004 | 127 | 30 | 69 | 185 |
| 2005 | 143 | 30 | 84 | 202 |
| 2006 | 161 | 29 | 105 | 217 |
| 2007 | 181 | 26 | 130 | 232 |
| 2008 | 200 | 24 | 152 | 248 |
| 2009 | 218 | 25 | 169 | 267 |
| 2010 | 233 | 27 | 179 | 286 |
| 2011 | 243 | 29 | 185 | 300 |
| 2012 | 247 | 30 | 189 | 305 |
| 2013 | 246 | 28 | 190 | 301 |
| 2014 | 240 | 26 | 189 | 291 |
| 2015 | 231 | 25 | 183 | 279 |
| 2016 | 220 | 26 | 169 | 270 |
| 2017 | 206 | 30 | 147 | 266 |
| 2018 | 192 | 38 | 118 | 266 |
| 2019 | 178 | 47 | 86 | 269 |
| 2020 | 163 | 57 | 52 | 274 |
| 2021 | 149 | 67 | 18 | 280 |
| 2022 | 134 | 77 | -17 | 286 |
| 2023 | 120 | 88 | -53 | 292 |
| 2024 | 105 | 99 | -88 | 299 |
| 2025 | 91 | 110 | -124 | 306 |
| 2026 | 77 | 121 | -160 | 313 |
| 2027 | 62 | 132 | -196 | 320 |
| 2028 | 48 | 143 | -232 | 327 |
| 2029 | 33 | 154 | -268 | 334 |
| 2030 | 19 | 165 | -304 | 342 |

| **Ischaemic heart disease** | | | | |
| --- | --- | --- | --- | --- |
| **Year** | **Mean** | **Standard Error of Mean** | **Min** | **Max** |
| 1990 | 465 | 86 | 296 | 633 |
| 1991 | 458 | 70 | 322 | 595 |
| 1992 | 452 | 56 | 342 | 562 |
| 1993 | 447 | 47 | 354 | 540 |
| 1994 | 443 | 45 | 355 | 531 |
| 1995 | 441 | 47 | 348 | 534 |
| 1996 | 442 | 51 | 342 | 543 |
| 1997 | 447 | 54 | 341 | 553 |
| 1998 | 456 | 54 | 350 | 561 |
| 1999 | 469 | 51 | 370 | 568 |
| 2000 | 486 | 46 | 395 | 577 |
| 2001 | 504 | 45 | 416 | 591 |
| 2002 | 521 | 46 | 430 | 612 |
| 2003 | 536 | 51 | 437 | 635 |
| 2004 | 546 | 55 | 439 | 654 |
| 2005 | 551 | 56 | 441 | 660 |
| 2006 | 548 | 53 | 444 | 652 |
| 2007 | 538 | 48 | 444 | 633 |
| 2008 | 524 | 45 | 436 | 613 |
| 2009 | 507 | 46 | 417 | 598 |
| 2010 | 489 | 50 | 390 | 588 |
| 2011 | 471 | 54 | 364 | 577 |
| 2012 | 454 | 55 | 346 | 562 |
| 2013 | 439 | 53 | 336 | 542 |
| 2014 | 426 | 48 | 331 | 521 |
| 2015 | 414 | 45 | 325 | 503 |
| 2016 | 402 | 47 | 309 | 495 |
| 2017 | 392 | 56 | 282 | 501 |
| 2018 | 381 | 70 | 244 | 518 |
| 2019 | 370 | 87 | 201 | 540 |
| 2020 | 360 | 105 | 155 | 565 |
| 2021 | 349 | 124 | 107 | 592 |
| 2022 | 339 | 143 | 58 | 620 |
| 2023 | 328 | 163 | 9 | 648 |
| 2024 | 318 | 183 | -41 | 676 |
| 2025 | 307 | 203 | -91 | 705 |
| 2026 | 297 | 223 | -141 | 735 |
| 2027 | 286 | 244 | -191 | 764 |
| 2028 | 276 | 264 | -242 | 794 |
| 2029 | 265 | 285 | -292 | 823 |
| 2030 | 255 | 305 | -343 | 853 |

| **Kidney cancer** | | | | |
| --- | --- | --- | --- | --- |
| **Year** | **Mean** | **Standard Error of Mean** | **Min** | **Max** |
| 1990 | -2 | 15 | -31 | 26 |
| 1991 | -1 | 12 | -25 | 22 |
| 1992 | -1 | 10 | -19 | 18 |
| 1993 | 0 | 8 | -16 | 16 |
| 1994 | 1 | 8 | -14 | 16 |
| 1995 | 2 | 8 | -14 | 17 |
| 1996 | 3 | 9 | -14 | 20 |
| 1997 | 4 | 9 | -14 | 22 |
| 1998 | 6 | 9 | -12 | 24 |
| 1999 | 8 | 9 | -9 | 25 |
| 2000 | 10 | 8 | -5 | 26 |
| 2001 | 12 | 8 | -3 | 27 |
| 2002 | 14 | 8 | -1 | 30 |
| 2003 | 16 | 9 | -1 | 33 |
| 2004 | 17 | 9 | -1 | 36 |
| 2005 | 18 | 9 | -1 | 37 |
| 2006 | 18 | 9 | 0 | 36 |
| 2007 | 17 | 8 | 1 | 33 |
| 2008 | 17 | 8 | 2 | 32 |
| 2009 | 16 | 8 | 1 | 31 |
| 2010 | 15 | 9 | -1 | 32 |
| 2011 | 15 | 9 | -3 | 34 |
| 2012 | 16 | 9 | -2 | 35 |
| 2013 | 18 | 9 | 0 | 35 |
| 2014 | 20 | 8 | 4 | 36 |
| 2015 | 23 | 8 | 8 | 38 |
| 2016 | 26 | 8 | 10 | 42 |
| 2017 | 29 | 10 | 10 | 48 |
| 2018 | 32 | 12 | 9 | 56 |
| 2019 | 36 | 15 | 7 | 65 |
| 2020 | 39 | 18 | 4 | 74 |
| 2021 | 43 | 21 | 1 | 84 |
| 2022 | 46 | 24 | -2 | 94 |
| 2023 | 49 | 28 | -5 | 104 |
| 2024 | 53 | 31 | -8 | 114 |
| 2025 | 56 | 34 | -11 | 124 |
| 2026 | 60 | 38 | -15 | 134 |
| 2027 | 63 | 41 | -18 | 144 |
| 2028 | 66 | 45 | -21 | 154 |
| 2029 | 70 | 48 | -25 | 164 |
| 2030 | 73 | 52 | -28 | 175 |

| **Leukaemia** | | | | |
| --- | --- | --- | --- | --- |
| **Year** | **Mean** | **Standard Error of Mean** | **Min** | **Max** |
| 1990 | 109 | 43 | 25 | 192 |
| 1991 | 115 | 34 | 48 | 183 |
| 1992 | 122 | 28 | 68 | 177 |
| 1993 | 129 | 23 | 83 | 175 |
| 1994 | 135 | 22 | 91 | 178 |
| 1995 | 139 | 23 | 93 | 185 |
| 1996 | 143 | 25 | 93 | 193 |
| 1997 | 145 | 27 | 92 | 197 |
| 1998 | 145 | 27 | 93 | 197 |
| 1999 | 143 | 25 | 94 | 192 |
| 2000 | 139 | 23 | 94 | 184 |
| 2001 | 134 | 22 | 91 | 177 |
| 2002 | 129 | 23 | 84 | 174 |
| 2003 | 124 | 25 | 75 | 173 |
| 2004 | 120 | 27 | 67 | 173 |
| 2005 | 118 | 28 | 63 | 172 |
| 2006 | 117 | 26 | 66 | 168 |
| 2007 | 118 | 24 | 71 | 165 |
| 2008 | 119 | 22 | 75 | 163 |
| 2009 | 121 | 23 | 76 | 166 |
| 2010 | 121 | 25 | 72 | 170 |
| 2011 | 121 | 27 | 68 | 173 |
| 2012 | 118 | 27 | 64 | 171 |
| 2013 | 113 | 26 | 62 | 164 |
| 2014 | 107 | 24 | 60 | 154 |
| 2015 | 100 | 23 | 56 | 144 |
| 2016 | 92 | 24 | 45 | 138 |
| 2017 | 83 | 28 | 28 | 137 |
| 2018 | 73 | 35 | 5 | 141 |
| 2019 | 64 | 43 | -20 | 148 |
| 2020 | 55 | 52 | -47 | 156 |
| 2021 | 45 | 61 | -75 | 166 |
| 2022 | 36 | 71 | -103 | 175 |
| 2023 | 27 | 81 | -131 | 185 |
| 2024 | 18 | 91 | -160 | 195 |
| 2025 | 8 | 101 | -189 | 205 |
| 2026 | -1 | 111 | -218 | 216 |
| 2027 | -10 | 121 | -247 | 226 |
| 2028 | -20 | 131 | -276 | 237 |
| 2029 | -29 | 141 | -305 | 247 |
| 2030 | -38 | 151 | -335 | 258 |

| **Lower respiratory infections** | | | | |
| --- | --- | --- | --- | --- |
| **Year** | **Mean** | **Standard Error of Mean** | **Min** | **Max** |
| 1990 | 141934 | 2148 | 137724 | 146143 |
| 1991 | 139691 | 1743 | 136275 | 143106 |
| 1992 | 137464 | 1404 | 134713 | 140216 |
| 1993 | 135336 | 1185 | 133014 | 137659 |
| 1994 | 133405 | 1122 | 131205 | 135605 |
| 1995 | 131768 | 1183 | 129450 | 134087 |
| 1996 | 130525 | 1284 | 128009 | 133041 |
| 1997 | 129773 | 1353 | 127122 | 132424 |
| 1998 | 129611 | 1348 | 126970 | 132253 |
| 1999 | 130063 | 1266 | 127583 | 132544 |
| 2000 | 130859 | 1163 | 128579 | 133139 |
| 2001 | 131654 | 1115 | 129469 | 133838 |
| 2002 | 132102 | 1160 | 129830 | 134375 |
| 2003 | 131861 | 1268 | 129375 | 134347 |
| 2004 | 130585 | 1371 | 127899 | 133271 |
| 2005 | 127929 | 1399 | 125188 | 130671 |
| 2006 | 123709 | 1325 | 121113 | 126306 |
| 2007 | 118380 | 1206 | 116016 | 120743 |
| 2008 | 112554 | 1130 | 110339 | 114769 |
| 2009 | 106847 | 1157 | 104579 | 109115 |
| 2010 | 101872 | 1262 | 99399 | 104345 |
| 2011 | 98244 | 1360 | 95579 | 100910 |
| 2012 | 96428 | 1378 | 93727 | 99130 |
| 2013 | 96293 | 1315 | 93716 | 98870 |
| 2014 | 97558 | 1210 | 95187 | 99929 |
| 2015 | 99943 | 1137 | 97715 | 102172 |
| 2016 | 103169 | 1188 | 100842 | 105497 |
| 2017 | 106956 | 1404 | 104204 | 109707 |
| 2018 | 111022 | 1750 | 107592 | 114451 |
| 2019 | 115135 | 2167 | 110887 | 119382 |
| 2020 | 119248 | 2621 | 114110 | 124386 |
| 2021 | 123361 | 3096 | 117292 | 129429 |
| 2022 | 127474 | 3584 | 120450 | 134497 |
| 2023 | 131587 | 4079 | 123592 | 139581 |
| 2024 | 135700 | 4579 | 126724 | 144675 |
| 2025 | 139813 | 5084 | 129848 | 149777 |
| 2026 | 143925 | 5591 | 132967 | 154884 |
| 2027 | 148038 | 6101 | 136081 | 159995 |
| 2028 | 152151 | 6612 | 139193 | 165110 |
| 2029 | 156264 | 7124 | 142302 | 170227 |
| 2030 | 160377 | 7637 | 145409 | 175345 |

| **Malaria** | | | | |
| --- | --- | --- | --- | --- |
| **Year** | **Mean** | **Standard Error of Mean** | **Min** | **Max** |
| 1990 | 44069 | 851 | 42401 | 45736 |
| 1991 | 41395 | 690 | 40042 | 42748 |
| 1992 | 38727 | 556 | 37637 | 39817 |
| 1993 | 36097 | 469 | 35177 | 37017 |
| 1994 | 33542 | 445 | 32670 | 34413 |
| 1995 | 31099 | 469 | 30181 | 32018 |
| 1996 | 28807 | 509 | 27810 | 29804 |
| 1997 | 26703 | 536 | 25652 | 27753 |
| 1998 | 24823 | 534 | 23777 | 25870 |
| 1999 | 23215 | 501 | 22232 | 24198 |
| 2000 | 21955 | 461 | 21052 | 22859 |
| 2001 | 21130 | 442 | 20264 | 21995 |
| 2002 | 20824 | 459 | 19923 | 21724 |
| 2003 | 21123 | 503 | 20138 | 22108 |
| 2004 | 22114 | 543 | 21050 | 23179 |
| 2005 | 23882 | 554 | 22796 | 24968 |
| 2006 | 26428 | 525 | 25399 | 27456 |
| 2007 | 29411 | 478 | 28475 | 30348 |
| 2008 | 32409 | 448 | 31532 | 33287 |
| 2009 | 34997 | 458 | 34098 | 35895 |
| 2010 | 36750 | 500 | 35770 | 37729 |
| 2011 | 37244 | 539 | 36188 | 38300 |
| 2012 | 36165 | 546 | 35095 | 37236 |
| 2013 | 33645 | 521 | 32624 | 34666 |
| 2014 | 29922 | 479 | 28982 | 30861 |
| 2015 | 25237 | 450 | 24354 | 26120 |
| 2016 | 19831 | 470 | 18908 | 20753 |
| 2017 | 13944 | 556 | 12853 | 15034 |
| 2018 | 7816 | 693 | 6457 | 9175 |
| 2019 | 1648 | 859 | -34 | 3331 |
| 2020 | -4519 | 1039 | -6555 | -2484 |
| 2021 | -10687 | 1227 | -13091 | -8282 |
| 2022 | -16854 | 1420 | -19637 | -14072 |
| 2023 | -23022 | 1616 | -26189 | -19855 |
| 2024 | -29189 | 1814 | -32745 | -25633 |
| 2025 | -35357 | 2014 | -39305 | -31409 |
| 2026 | -41524 | 2215 | -45866 | -37183 |
| 2027 | -47692 | 2417 | -52429 | -42955 |
| 2028 | -53860 | 2619 | -58994 | -48726 |
| 2029 | -60027 | 2822 | -65559 | -54495 |
| 2030 | -66195 | 3026 | -72125 | -60265 |

| **Measles** | | | | |
| --- | --- | --- | --- | --- |
| **Year** | **Mean** | **Standard Error of Mean** | **Min** | **Max** |
| 1990 | 3574 | 185 | 3211 | 3936 |
| 1991 | 3506 | 150 | 3212 | 3800 |
| 1992 | 3438 | 121 | 3201 | 3675 |
| 1993 | 3370 | 102 | 3170 | 3569 |
| 1994 | 3300 | 97 | 3110 | 3489 |
| 1995 | 3228 | 102 | 3028 | 3427 |
| 1996 | 3153 | 111 | 2936 | 3370 |
| 1997 | 3075 | 116 | 2847 | 3303 |
| 1998 | 2993 | 116 | 2765 | 3220 |
| 1999 | 2906 | 109 | 2692 | 3119 |
| 2000 | 2812 | 100 | 2615 | 3008 |
| 2001 | 2709 | 96 | 2521 | 2897 |
| 2002 | 2595 | 100 | 2399 | 2791 |
| 2003 | 2468 | 109 | 2254 | 2682 |
| 2004 | 2326 | 118 | 2095 | 2558 |
| 2005 | 2168 | 120 | 1931 | 2404 |
| 2006 | 1992 | 114 | 1768 | 2215 |
| 2007 | 1806 | 104 | 1602 | 2010 |
| 2008 | 1620 | 97 | 1430 | 1811 |
| 2009 | 1444 | 100 | 1249 | 1640 |
| 2010 | 1287 | 109 | 1074 | 1500 |
| 2011 | 1159 | 117 | 929 | 1388 |
| 2012 | 1066 | 119 | 833 | 1299 |
| 2013 | 1006 | 113 | 784 | 1228 |
| 2014 | 973 | 104 | 769 | 1177 |
| 2015 | 962 | 98 | 770 | 1154 |
| 2016 | 967 | 102 | 767 | 1168 |
| 2017 | 983 | 121 | 747 | 1220 |
| 2018 | 1005 | 151 | 710 | 1301 |
| 2019 | 1028 | 187 | 662 | 1394 |
| 2020 | 1050 | 226 | 608 | 1493 |
| 2021 | 1073 | 267 | 550 | 1595 |
| 2022 | 1095 | 309 | 490 | 1700 |
| 2023 | 1118 | 351 | 429 | 1806 |
| 2024 | 1140 | 394 | 367 | 1913 |
| 2025 | 1163 | 438 | 305 | 2021 |
| 2026 | 1186 | 482 | 242 | 2129 |
| 2027 | 1208 | 525 | 178 | 2238 |
| 2028 | 1231 | 569 | 115 | 2347 |
| 2029 | 1253 | 614 | 51 | 2456 |
| 2030 | 1276 | 658 | -13 | 2565 |

| **Non-rheumatic heart disease** | | | | |
| --- | --- | --- | --- | --- |
| **Year** | **Mean** | **Standard Error of Mean** | **Min** | **Max** |
| 1990 | 23 | 29 | -34 | 81 |
| 1991 | 31 | 24 | -16 | 77 |
| 1992 | 38 | 19 | 1 | 76 |
| 1993 | 46 | 16 | 14 | 77 |
| 1994 | 52 | 15 | 22 | 82 |
| 1995 | 58 | 16 | 27 | 90 |
| 1996 | 63 | 17 | 29 | 98 |
| 1997 | 67 | 18 | 31 | 103 |
| 1998 | 70 | 18 | 34 | 106 |
| 1999 | 70 | 17 | 37 | 104 |
| 2000 | 70 | 16 | 39 | 101 |
| 2001 | 68 | 15 | 38 | 98 |
| 2002 | 66 | 16 | 35 | 97 |
| 2003 | 63 | 17 | 29 | 97 |
| 2004 | 60 | 19 | 24 | 97 |
| 2005 | 57 | 19 | 20 | 95 |
| 2006 | 55 | 18 | 20 | 91 |
| 2007 | 54 | 16 | 21 | 86 |
| 2008 | 53 | 15 | 22 | 83 |
| 2009 | 52 | 16 | 21 | 83 |
| 2010 | 52 | 17 | 18 | 86 |
| 2011 | 53 | 19 | 17 | 89 |
| 2012 | 54 | 19 | 17 | 91 |
| 2013 | 56 | 18 | 21 | 91 |
| 2014 | 58 | 16 | 26 | 91 |
| 2015 | 61 | 15 | 30 | 91 |
| 2016 | 64 | 16 | 32 | 95 |
| 2017 | 67 | 19 | 29 | 104 |
| 2018 | 70 | 24 | 23 | 117 |
| 2019 | 73 | 30 | 15 | 131 |
| 2020 | 76 | 36 | 6 | 146 |
| 2021 | 79 | 42 | -3 | 162 |
| 2022 | 83 | 49 | -13 | 178 |
| 2023 | 86 | 56 | -23 | 195 |
| 2024 | 89 | 62 | -33 | 211 |
| 2025 | 92 | 69 | -44 | 228 |
| 2026 | 95 | 76 | -54 | 245 |
| 2027 | 98 | 83 | -65 | 261 |
| 2028 | 102 | 90 | -75 | 278 |
| 2029 | 105 | 97 | -86 | 295 |
| 2030 | 108 | 104 | -96 | 312 |

| **Other neoplasms** | | | | |
| --- | --- | --- | --- | --- |
| **Year** | **Mean** | **Standard Error of Mean** | **Min** | **Max** |
| 1990 | 115370 | 2096 | 111263 | 119478 |
| 1991 | 113832 | 1700 | 110500 | 117165 |
| 1992 | 112312 | 1370 | 109628 | 114997 |
| 1993 | 110901 | 1156 | 108635 | 113167 |
| 1994 | 109707 | 1095 | 107560 | 111853 |
| 1995 | 108839 | 1154 | 106576 | 111101 |
| 1996 | 108405 | 1252 | 105950 | 110860 |
| 1997 | 108515 | 1320 | 105928 | 111101 |
| 1998 | 109276 | 1315 | 106699 | 111853 |
| 1999 | 110747 | 1235 | 108326 | 113167 |
| 2000 | 112778 | 1135 | 110553 | 115003 |
| 2001 | 115171 | 1088 | 113039 | 117302 |
| 2002 | 117725 | 1131 | 115508 | 119943 |
| 2003 | 120242 | 1238 | 117816 | 122667 |
| 2004 | 122521 | 1337 | 119900 | 125141 |
| 2005 | 124363 | 1365 | 121688 | 127038 |
| 2006 | 125614 | 1293 | 123081 | 128148 |
| 2007 | 126306 | 1176 | 124001 | 128612 |
| 2008 | 126517 | 1103 | 124356 | 128678 |
| 2009 | 126323 | 1129 | 124110 | 128536 |
| 2010 | 125802 | 1231 | 123389 | 128215 |
| 2011 | 125032 | 1327 | 122431 | 127632 |
| 2012 | 124080 | 1345 | 121444 | 126716 |
| 2013 | 122981 | 1283 | 120467 | 125495 |
| 2014 | 121759 | 1180 | 119446 | 124072 |
| 2015 | 120438 | 1109 | 118264 | 122613 |
| 2016 | 119044 | 1159 | 116773 | 121315 |
| 2017 | 117601 | 1370 | 114916 | 120285 |
| 2018 | 116133 | 1707 | 112787 | 119479 |
| 2019 | 114661 | 2114 | 110516 | 118805 |
| 2020 | 113189 | 2558 | 108176 | 118201 |
| 2021 | 111716 | 3021 | 105796 | 117637 |
| 2022 | 110244 | 3496 | 103392 | 117097 |
| 2023 | 108772 | 3980 | 100972 | 116572 |
| 2024 | 107300 | 4468 | 98543 | 116057 |
| 2025 | 105828 | 4960 | 96106 | 115550 |
| 2026 | 104356 | 5455 | 93664 | 115048 |
| 2027 | 102884 | 5952 | 91218 | 114550 |
| 2028 | 101412 | 6451 | 88768 | 114055 |
| 2029 | 99939 | 6950 | 86317 | 113562 |
| 2030 | 98467 | 7451 | 83863 | 113071 |

| **Ovarian cancer** | | | | |
| --- | --- | --- | --- | --- |
| **Year** | **Mean** | **Standard Error of Mean** | **Min** | **Max** |
| 1990 | 90 | 48 | -4 | 184 |
| 1991 | 96 | 39 | 20 | 173 |
| 1992 | 103 | 31 | 41 | 164 |
| 1993 | 109 | 27 | 57 | 161 |
| 1994 | 115 | 25 | 65 | 164 |
| 1995 | 120 | 26 | 68 | 172 |
| 1996 | 125 | 29 | 69 | 181 |
| 1997 | 129 | 30 | 70 | 188 |
| 1998 | 132 | 30 | 73 | 191 |
| 1999 | 135 | 28 | 79 | 190 |
| 2000 | 136 | 26 | 85 | 187 |
| 2001 | 138 | 25 | 89 | 187 |
| 2002 | 139 | 26 | 88 | 190 |
| 2003 | 141 | 28 | 85 | 197 |
| 2004 | 144 | 31 | 83 | 204 |
| 2005 | 147 | 31 | 86 | 209 |
| 2006 | 153 | 30 | 94 | 211 |
| 2007 | 159 | 27 | 106 | 212 |
| 2008 | 166 | 25 | 116 | 215 |
| 2009 | 173 | 26 | 122 | 223 |
| 2010 | 179 | 28 | 124 | 235 |
| 2011 | 185 | 30 | 125 | 245 |
| 2012 | 189 | 31 | 129 | 250 |
| 2013 | 193 | 29 | 135 | 250 |
| 2014 | 195 | 27 | 142 | 248 |
| 2015 | 196 | 25 | 147 | 246 |
| 2016 | 197 | 27 | 145 | 249 |
| 2017 | 198 | 31 | 136 | 259 |
| 2018 | 198 | 39 | 122 | 275 |
| 2019 | 199 | 48 | 104 | 294 |
| 2020 | 199 | 59 | 84 | 314 |
| 2021 | 199 | 69 | 63 | 335 |
| 2022 | 200 | 80 | 42 | 357 |
| 2023 | 200 | 91 | 21 | 379 |
| 2024 | 200 | 102 | -1 | 401 |
| 2025 | 201 | 114 | -22 | 424 |
| 2026 | 201 | 125 | -44 | 446 |
| 2027 | 201 | 137 | -66 | 469 |
| 2028 | 202 | 148 | -88 | 491 |
| 2029 | 202 | 159 | -111 | 514 |
| 2030 | 202 | 171 | -133 | 537 |

| **Sexually transmitted infections (excluding HIV/AIDs)** | | | | |
| --- | --- | --- | --- | --- |
| **Year** | **Mean** | **Standard Error of Mean** | **Min** | **Max** |
| 1990 | 538077 | 8006 | 522386 | 553769 |
| 1991 | 530665 | 6496 | 517934 | 543396 |
| 1992 | 523329 | 5232 | 513074 | 533584 |
| 1993 | 516453 | 4416 | 507797 | 525109 |
| 1994 | 510495 | 4184 | 502295 | 518696 |
| 1995 | 505916 | 4410 | 497272 | 514559 |
| 1996 | 503173 | 4785 | 493795 | 512551 |
| 1997 | 502728 | 5042 | 492847 | 512609 |
| 1998 | 505038 | 5024 | 495192 | 514885 |
| 1999 | 510368 | 4718 | 501122 | 519614 |
| 2000 | 518192 | 4336 | 509693 | 526691 |
| 2001 | 527789 | 4155 | 519645 | 535933 |
| 2002 | 538440 | 4322 | 529968 | 546912 |
| 2003 | 549423 | 4728 | 540156 | 558690 |
| 2004 | 560017 | 5109 | 550004 | 570030 |
| 2005 | 569502 | 5214 | 559282 | 579721 |
| 2006 | 577273 | 4938 | 567594 | 586952 |
| 2007 | 583199 | 4495 | 574389 | 592009 |
| 2008 | 587263 | 4213 | 579007 | 595520 |
| 2009 | 589451 | 4313 | 580997 | 597904 |
| 2010 | 589746 | 4703 | 580528 | 598963 |
| 2011 | 588132 | 5069 | 578198 | 598067 |
| 2012 | 584644 | 5138 | 574573 | 594714 |
| 2013 | 579506 | 4901 | 569901 | 589111 |
| 2014 | 572995 | 4509 | 564157 | 581833 |
| 2015 | 565384 | 4238 | 557077 | 573691 |
| 2016 | 556949 | 4426 | 548273 | 565625 |
| 2017 | 547965 | 5233 | 537709 | 558221 |
| 2018 | 538705 | 6523 | 525921 | 551489 |
| 2019 | 529400 | 8078 | 513567 | 545233 |
| 2020 | 520095 | 9771 | 500943 | 539246 |
| 2021 | 510789 | 11541 | 488169 | 533410 |
| 2022 | 501484 | 13358 | 475304 | 527665 |
| 2023 | 492179 | 15204 | 462380 | 521978 |
| 2024 | 482874 | 17070 | 449417 | 516330 |
| 2025 | 473569 | 18951 | 436426 | 510711 |
| 2026 | 464263 | 20841 | 423415 | 505111 |
| 2027 | 454958 | 22740 | 410388 | 499527 |
| 2028 | 445653 | 24644 | 397350 | 493955 |
| 2029 | 436347 | 26554 | 384303 | 488392 |
| 2030 | 427042 | 28467 | 371249 | 482836 |

| **Stomach cancer** | | | | |
| --- | --- | --- | --- | --- |
| **Year** | **Mean** | **Standard Error of Mean** | **Min** | **Max** |
| 1990 | 29 | 28 | -25 | 83 |
| 1991 | 36 | 22 | -8 | 79 |
| 1992 | 42 | 18 | 7 | 78 |
| 1993 | 49 | 15 | 19 | 79 |
| 1994 | 55 | 14 | 27 | 83 |
| 1995 | 61 | 15 | 31 | 90 |
| 1996 | 65 | 16 | 33 | 97 |
| 1997 | 69 | 17 | 35 | 103 |
| 1998 | 71 | 17 | 37 | 105 |
| 1999 | 72 | 16 | 40 | 104 |
| 2000 | 72 | 15 | 42 | 101 |
| 2001 | 70 | 14 | 42 | 98 |
| 2002 | 67 | 15 | 38 | 97 |
| 2003 | 64 | 16 | 32 | 96 |
| 2004 | 60 | 18 | 26 | 95 |
| 2005 | 56 | 18 | 20 | 91 |
| 2006 | 51 | 17 | 17 | 84 |
| 2007 | 46 | 15 | 16 | 76 |
| 2008 | 41 | 15 | 13 | 70 |
| 2009 | 37 | 15 | 8 | 67 |
| 2010 | 35 | 16 | 3 | 66 |
| 2011 | 33 | 17 | -1 | 67 |
| 2012 | 33 | 18 | -2 | 67 |
| 2013 | 34 | 17 | 1 | 67 |
| 2014 | 36 | 16 | 6 | 66 |
| 2015 | 39 | 15 | 10 | 68 |
| 2016 | 43 | 15 | 13 | 73 |
| 2017 | 47 | 18 | 12 | 82 |
| 2018 | 52 | 22 | 8 | 96 |
| 2019 | 56 | 28 | 2 | 111 |
| 2020 | 61 | 34 | -5 | 127 |
| 2021 | 65 | 40 | -13 | 143 |
| 2022 | 70 | 46 | -20 | 160 |
| 2023 | 74 | 52 | -28 | 177 |
| 2024 | 79 | 59 | -36 | 194 |
| 2025 | 83 | 65 | -44 | 211 |
| 2026 | 88 | 72 | -53 | 229 |
| 2027 | 92 | 78 | -61 | 246 |
| 2028 | 97 | 85 | -69 | 263 |
| 2029 | 102 | 91 | -78 | 281 |
| 2030 | 106 | 98 | -86 | 298 |

| **Stroke** | | | | |
| --- | --- | --- | --- | --- |
| **Year** | **Mean** | **Standard Error of Mean** | **Min** | **Max** |
| 1990 | 2986 | 234 | 2528 | 3444 |
| 1991 | 2957 | 189 | 2585 | 3328 |
| 1992 | 2928 | 153 | 2629 | 3227 |
| 1993 | 2903 | 129 | 2651 | 3156 |
| 1994 | 2885 | 122 | 2646 | 3124 |
| 1995 | 2877 | 129 | 2625 | 3129 |
| 1996 | 2883 | 140 | 2610 | 3157 |
| 1997 | 2906 | 147 | 2618 | 3195 |
| 1998 | 2950 | 147 | 2663 | 3238 |
| 1999 | 3017 | 138 | 2747 | 3287 |
| 2000 | 3102 | 127 | 2854 | 3350 |
| 2001 | 3199 | 121 | 2962 | 3437 |
| 2002 | 3304 | 126 | 3057 | 3552 |
| 2003 | 3411 | 138 | 3141 | 3681 |
| 2004 | 3514 | 149 | 3222 | 3806 |
| 2005 | 3607 | 152 | 3308 | 3905 |
| 2006 | 3685 | 144 | 3403 | 3967 |
| 2007 | 3748 | 131 | 3491 | 4005 |
| 2008 | 3794 | 123 | 3553 | 4035 |
| 2009 | 3824 | 126 | 3577 | 4070 |
| 2010 | 3835 | 137 | 3567 | 4104 |
| 2011 | 3829 | 148 | 3540 | 4119 |
| 2012 | 3805 | 150 | 3512 | 4099 |
| 2013 | 3765 | 143 | 3485 | 4046 |
| 2014 | 3712 | 132 | 3454 | 3970 |
| 2015 | 3648 | 124 | 3406 | 3891 |
| 2016 | 3576 | 129 | 3323 | 3830 |
| 2017 | 3499 | 153 | 3200 | 3799 |
| 2018 | 3420 | 190 | 3047 | 3792 |
| 2019 | 3339 | 236 | 2877 | 3801 |
| 2020 | 3259 | 285 | 2700 | 3818 |
| 2021 | 3179 | 337 | 2519 | 3839 |
| 2022 | 3099 | 390 | 2335 | 3862 |
| 2023 | 3019 | 444 | 2149 | 3888 |
| 2024 | 2938 | 498 | 1962 | 3914 |
| 2025 | 2858 | 553 | 1775 | 3942 |
| 2026 | 2778 | 608 | 1586 | 3970 |
| 2027 | 2698 | 663 | 1397 | 3998 |
| 2028 | 2617 | 719 | 1208 | 4027 |
| 2029 | 2537 | 775 | 1019 | 4056 |
| 2030 | 2457 | 830 | 829 | 4085 |

| **Tuberculosis** | | | | |
| --- | --- | --- | --- | --- |
| **Year** | **Mean** | **Standard Error of Mean** | **Min** | **Max** |
| 1990 | 17784 | 372 | 17056 | 18513 |
| 1991 | 16640 | 302 | 16049 | 17231 |
| 1992 | 15499 | 243 | 15023 | 15975 |
| 1993 | 14378 | 205 | 13976 | 14780 |
| 1994 | 13299 | 194 | 12918 | 13679 |
| 1995 | 12280 | 205 | 11879 | 12682 |
| 1996 | 11343 | 222 | 10908 | 11779 |
| 1997 | 10509 | 234 | 10050 | 10968 |
| 1998 | 9797 | 233 | 9339 | 10254 |
| 1999 | 9219 | 219 | 8790 | 9649 |
| 2000 | 8757 | 201 | 8362 | 9152 |
| 2001 | 8381 | 193 | 8003 | 8760 |
| 2002 | 8065 | 201 | 7671 | 8458 |
| 2003 | 7778 | 220 | 7348 | 8209 |
| 2004 | 7495 | 237 | 7030 | 7960 |
| 2005 | 7186 | 242 | 6712 | 7661 |
| 2006 | 6831 | 229 | 6382 | 7281 |
| 2007 | 6439 | 209 | 6029 | 6848 |
| 2008 | 6025 | 196 | 5641 | 6408 |
| 2009 | 5605 | 200 | 5213 | 5998 |
| 2010 | 5197 | 218 | 4769 | 5625 |
| 2011 | 4815 | 235 | 4354 | 5276 |
| 2012 | 4472 | 239 | 4005 | 4940 |
| 2013 | 4166 | 228 | 3720 | 4612 |
| 2014 | 3891 | 209 | 3480 | 4301 |
| 2015 | 3640 | 197 | 3254 | 4026 |
| 2016 | 3407 | 206 | 3004 | 3810 |
| 2017 | 3187 | 243 | 2711 | 3663 |
| 2018 | 2973 | 303 | 2379 | 3566 |
| 2019 | 2759 | 375 | 2024 | 3495 |
| 2020 | 2546 | 454 | 1657 | 3435 |
| 2021 | 2333 | 536 | 1282 | 3383 |
| 2022 | 2119 | 620 | 904 | 3335 |
| 2023 | 1906 | 706 | 522 | 3290 |
| 2024 | 1693 | 793 | 139 | 3246 |
| 2025 | 1479 | 880 | -245 | 3204 |
| 2026 | 1266 | 968 | -631 | 3163 |
| 2027 | 1053 | 1056 | -1017 | 3123 |
| 2028 | 840 | 1144 | -1404 | 3083 |
| 2029 | 626 | 1233 | -1791 | 3043 |
| 2030 | 413 | 1322 | -2178 | 3004 |

| **Thyroid cancer** | |  |  |  |
| --- | --- | --- | --- | --- |
| **Year** | **Mean** | **Standard Error of Mean** | **Min** | **Max** |
| 1990 | 19 | 33 | -45 | 83 |
| 1991 | 28 | 26 | -23 | 80 |
| 1992 | 37 | 21 | -4 | 79 |
| 1993 | 46 | 18 | 11 | 82 |
| 1994 | 55 | 17 | 22 | 88 |
| 1995 | 63 | 18 | 27 | 98 |
| 1996 | 69 | 19 | 31 | 107 |
| 1997 | 75 | 20 | 35 | 115 |
| 1998 | 79 | 20 | 39 | 118 |
| 1999 | 81 | 19 | 43 | 118 |
| 2000 | 82 | 18 | 47 | 116 |
| 2001 | 82 | 17 | 48 | 115 |
| 2002 | 81 | 18 | 46 | 115 |
| 2003 | 80 | 19 | 42 | 118 |
| 2004 | 79 | 21 | 39 | 120 |
| 2005 | 79 | 21 | 38 | 121 |
| 2006 | 80 | 20 | 40 | 119 |
| 2007 | 81 | 18 | 45 | 117 |
| 2008 | 82 | 17 | 49 | 116 |
| 2009 | 84 | 18 | 50 | 118 |
| 2010 | 85 | 19 | 47 | 122 |
| 2011 | 85 | 21 | 45 | 125 |
| 2012 | 84 | 21 | 43 | 125 |
| 2013 | 82 | 20 | 43 | 121 |
| 2014 | 79 | 18 | 43 | 115 |
| 2015 | 75 | 17 | 42 | 109 |
| 2016 | 71 | 18 | 36 | 107 |
| 2017 | 67 | 21 | 25 | 109 |
| 2018 | 62 | 26 | 11 | 114 |
| 2019 | 58 | 33 | -6 | 122 |
| 2020 | 53 | 40 | -24 | 131 |
| 2021 | 49 | 47 | -43 | 141 |
| 2022 | 44 | 54 | -62 | 150 |
| 2023 | 40 | 62 | -81 | 161 |
| 2024 | 35 | 69 | -101 | 171 |
| 2025 | 30 | 77 | -120 | 181 |
| 2026 | 26 | 85 | -140 | 192 |
| 2027 | 21 | 92 | -160 | 202 |
| 2028 | 17 | 100 | -179 | 213 |
| 2029 | 12 | 108 | -199 | 223 |
| 2030 | 7 | 116 | -219 | 234 |

| **Tracheal, bronchial and lung cancer** | | | | |
| --- | --- | --- | --- | --- |
| **Year** | **Mean** | **Standard Error of Mean** | **Min** | **Max** |
| 1990 | 25 | 33 | -40 | 91 |
| 1991 | 33 | 27 | -20 | 87 |
| 1992 | 42 | 22 | -1 | 84 |
| 1993 | 50 | 18 | 14 | 86 |
| 1994 | 57 | 17 | 23 | 92 |
| 1995 | 64 | 18 | 28 | 100 |
| 1996 | 70 | 20 | 31 | 110 |
| 1997 | 75 | 21 | 34 | 117 |
| 1998 | 79 | 21 | 38 | 120 |
| 1999 | 81 | 20 | 43 | 120 |
| 2000 | 82 | 18 | 47 | 118 |
| 2001 | 83 | 17 | 49 | 117 |
| 2002 | 83 | 18 | 47 | 118 |
| 2003 | 82 | 20 | 44 | 121 |
| 2004 | 82 | 21 | 41 | 124 |
| 2005 | 83 | 22 | 40 | 126 |
| 2006 | 85 | 21 | 44 | 125 |
| 2007 | 87 | 19 | 50 | 123 |
| 2008 | 89 | 18 | 54 | 123 |
| 2009 | 91 | 18 | 56 | 126 |
| 2010 | 92 | 20 | 54 | 131 |
| 2011 | 92 | 21 | 51 | 134 |
| 2012 | 91 | 21 | 49 | 133 |
| 2013 | 89 | 20 | 49 | 129 |
| 2014 | 85 | 19 | 48 | 122 |
| 2015 | 80 | 18 | 46 | 115 |
| 2016 | 75 | 18 | 39 | 111 |
| 2017 | 70 | 22 | 27 | 112 |
| 2018 | 64 | 27 | 10 | 117 |
| 2019 | 58 | 34 | -8 | 124 |
| 2020 | 52 | 41 | -28 | 132 |
| 2021 | 46 | 48 | -48 | 140 |
| 2022 | 40 | 56 | -69 | 149 |
| 2023 | 34 | 63 | -90 | 159 |
| 2024 | 28 | 71 | -111 | 168 |
| 2025 | 23 | 79 | -132 | 177 |
| 2026 | 17 | 87 | -154 | 187 |
| 2027 | 11 | 95 | -175 | 197 |
| 2028 | 5 | 103 | -196 | 206 |
| 2029 | -1 | 111 | -218 | 216 |
| 2030 | -7 | 119 | -240 | 226 |

| **Uterine cancer** | | | | |
| --- | --- | --- | --- | --- |
| **Year** | **Mean** | **Standard Error of Mean** | **Min** | **Max** |
| 1990 | 19 | 33 | -45 | 83 |
| 1991 | 28 | 27 | -24 | 80 |
| 1992 | 37 | 21 | -4 | 79 |
| 1993 | 47 | 18 | 11 | 82 |
| 1994 | 55 | 17 | 22 | 89 |
| 1995 | 63 | 18 | 28 | 98 |
| 1996 | 70 | 20 | 31 | 108 |
| 1997 | 75 | 21 | 35 | 115 |
| 1998 | 79 | 20 | 39 | 119 |
| 1999 | 81 | 19 | 43 | 119 |
| 2000 | 81 | 18 | 47 | 116 |
| 2001 | 81 | 17 | 48 | 114 |
| 2002 | 80 | 18 | 46 | 115 |
| 2003 | 79 | 19 | 41 | 117 |
| 2004 | 78 | 21 | 37 | 119 |
| 2005 | 78 | 21 | 37 | 120 |
| 2006 | 79 | 20 | 40 | 119 |
| 2007 | 81 | 18 | 45 | 117 |
| 2008 | 83 | 17 | 50 | 117 |
| 2009 | 85 | 18 | 51 | 120 |
| 2010 | 87 | 19 | 49 | 125 |
| 2011 | 88 | 21 | 47 | 128 |
| 2012 | 87 | 21 | 46 | 128 |
| 2013 | 85 | 20 | 46 | 124 |
| 2014 | 82 | 18 | 46 | 118 |
| 2015 | 79 | 17 | 45 | 113 |
| 2016 | 75 | 18 | 39 | 110 |
| 2017 | 70 | 21 | 28 | 112 |
| 2018 | 65 | 27 | 13 | 118 |
| 2019 | 61 | 33 | -4 | 125 |
| 2020 | 56 | 40 | -22 | 134 |
| 2021 | 51 | 47 | -41 | 143 |
| 2022 | 46 | 55 | -61 | 153 |
| 2023 | 41 | 62 | -80 | 163 |
| 2024 | 37 | 70 | -100 | 173 |
| 2025 | 32 | 77 | -120 | 183 |
| 2026 | 27 | 85 | -140 | 194 |
| 2027 | 22 | 93 | -159 | 204 |
| 2028 | 18 | 101 | -179 | 215 |
| 2029 | 13 | 108 | -200 | 225 |
| 2030 | 8 | 116 | -220 | 236 |

| **Vitamin A deficiency** | | | | |
| --- | --- | --- | --- | --- |
| **Year** | **Mean** | **Standard Error of Mean** | **Min** | **Max** |
| 1990 | 747118 | 6141 | 735081 | 759155 |
| 1991 | 705109 | 4983 | 695343 | 714875 |
| 1992 | 663235 | 4013 | 655368 | 671101 |
| 1993 | 622169 | 3388 | 615529 | 628809 |
| 1994 | 582720 | 3209 | 576430 | 589011 |
| 1995 | 545697 | 3383 | 539067 | 552327 |
| 1996 | 511908 | 3670 | 504714 | 519102 |
| 1997 | 482162 | 3867 | 474582 | 489741 |
| 1998 | 457266 | 3854 | 449714 | 464819 |
| 1999 | 437703 | 3619 | 430610 | 444795 |
| 2000 | 422637 | 3326 | 416117 | 429156 |
| 2001 | 410907 | 3187 | 404660 | 417155 |
| 2002 | 401353 | 3316 | 394855 | 407852 |
| 2003 | 392812 | 3627 | 385704 | 399921 |
| 2004 | 384123 | 3919 | 376443 | 391804 |
| 2005 | 374125 | 4000 | 366286 | 381964 |
| 2006 | 361943 | 3788 | 354518 | 369367 |
| 2007 | 347855 | 3448 | 341097 | 354612 |
| 2008 | 332426 | 3231 | 326092 | 338759 |
| 2009 | 316221 | 3308 | 309737 | 322706 |
| 2010 | 299806 | 3608 | 292735 | 306877 |
| 2011 | 283746 | 3888 | 276125 | 291367 |
| 2012 | 268490 | 3941 | 260765 | 276214 |
| 2013 | 254022 | 3759 | 246654 | 261389 |
| 2014 | 240211 | 3459 | 233431 | 246990 |
| 2015 | 226926 | 3251 | 220553 | 233298 |
| 2016 | 214035 | 3395 | 207380 | 220690 |
| 2017 | 201407 | 4014 | 193540 | 209274 |
| 2018 | 188910 | 5003 | 179104 | 198716 |
| 2019 | 176435 | 6197 | 164290 | 188581 |
| 2020 | 163961 | 7495 | 149270 | 178651 |
| 2021 | 151486 | 8853 | 134134 | 168838 |
| 2022 | 139011 | 10246 | 118929 | 159094 |
| 2023 | 126537 | 11662 | 103679 | 149395 |
| 2024 | 114062 | 13094 | 88398 | 139726 |
| 2025 | 101587 | 14537 | 73096 | 130079 |
| 2026 | 89112 | 15987 | 57779 | 120446 |
| 2027 | 76638 | 17443 | 42449 | 110826 |
| 2028 | 64163 | 18904 | 27111 | 101215 |
| 2029 | 51688 | 20369 | 11766 | 91610 |
| 2030 | 39214 | 21836 | -3584 | 82012 |
